# Binding Modes of Teixobactin to Lipid II: Molecular Dynamics Study

**DOI:** 10.1038/s41598-017-17606-5

**Published:** 2017-12-08

**Authors:** Yang Liu, Yaxin Liu, Mary B. Chan-Park, Yuguang Mu

**Affiliations:** 1 School of Biological Sciences, Nanyang Technological University (NTU), 60 Nanyang Drive, Singapore, 637551 Singapore; 2 School of Chemical and Biomedical Engineering, Nanyang Technological University (NTU), 62 Nanyang Drive, Singapore, 637459 Singapore; 3 Centre for Antimicrobial Bioengineering, NTU, 60 Nanyang Drive, Singapore, 637551 Singapore

## Abstract

Teixobactin (TXB) is a newly discovered antibiotic targeting the bacterial cell wall precursor Lipid II (L_II_). In the present work, four binding modes of TXB on L_II_ were identified by a contact-map based clustering method. The highly flexible binary complex ensemble was generated by parallel tempering metadynamics simulation in a well-tempered ensemble (PTMetaD-WTE). In agreement with experimental findings, the pyrophosphate group and the attached first sugar subunit of L_II_ are found to be the minimal motif for stable TXB binding. Three of the four binding modes involve the ring structure of TXB and have relatively higher binding affinities, indicating the importance of the ring motif of TXB in L_II_ recognition. TXB-L_II_ complexes with a ratio of 2:1 are also predicted with configurations such that the ring motif of two TXB molecules bound to the pyrophosphate-MurNAc moiety and the glutamic acid residue of one L_II_, respectively. Our findings disclose that the ring motif of TXB is critical to L_II_ binding and novel antibiotics can be designed based on its mimetics.

## Introduction

Lipid II (L_II_), one of the most essential intermediate products for the biosynthesis of bacterial cell walls, was recognized as an antibiotic target for decades^1^. Structurally, one L_II_ molecule is composed of one bactoprenol hydrocarbon chain (C55), which is embedded in the cell membrane, a disaccharide of N-acetylglucosamine (GlcNAc) and N-acetylmuramic acid (MurNAc), a penta-peptide attached to the MurNAc (typically with the sequence of L-alanyl-γ-D-glutamyl-L-lysyl-D-alanyl-D-alanine), and a pyrophosphate group (PP) linking the bactoprenol anchor and MurNAc^2,3^. The disaccharide and penta-peptide form the peptidoglycan subunit is further cross-linked through the penta-peptide to provide mechanical strength to the cell wall. L_II_ is critical to the cell wall synthetic pathway^4^: the molecule is first assembled to the C55 anchor in the cytoplasmic side of the plasma membrane, and then translocated to the periplasmic side where the pentapeptide-linked disaccharide is released from the anchor to build the cell wall. The remaining C55-PP is translocated back to the cytoplasmic side of the membrane by a not well characterized recycling pathway^5^. Thus, preventing the translocation of L_II_ is *bona fide* strategy to kill bacteria by interfering with a critical step in the cell wall synthetic pathway. Some antibiotics, such as vancomycin, nisin, ramoplanin and mannopeptimycin^2^ exert their antimicrobial activity by interacting with L_II_ synthesis.

The interaction between vancomycin and L_II_ was well investigated experimentally and computationally: vancomycin binds to L_II_ through five hydrogen bonds (H bonds) with the D-Ala-D-Ala segment of the penta-peptide of L_II_^6,7^. Several of these complexes can even form a super-complex through the D-Ala-D-Ala segment^8,9^_._ However, bacteria develop resistance to vancomycin by mutating the penta-peptide terminus from D-Ala-D-Ala to D-Ala-D-Lac. The binding affinity of vancomycin to the mutated L_II_ is 1000-fold lower mainly due to the introduction of a lone pair repulsion and the loss of central H bonds^10^. To counteract such effect, a vancomycin derivative was developed with similar binding affinity for both normal L_II_ and mutated L_II_.

**Figure 1:**
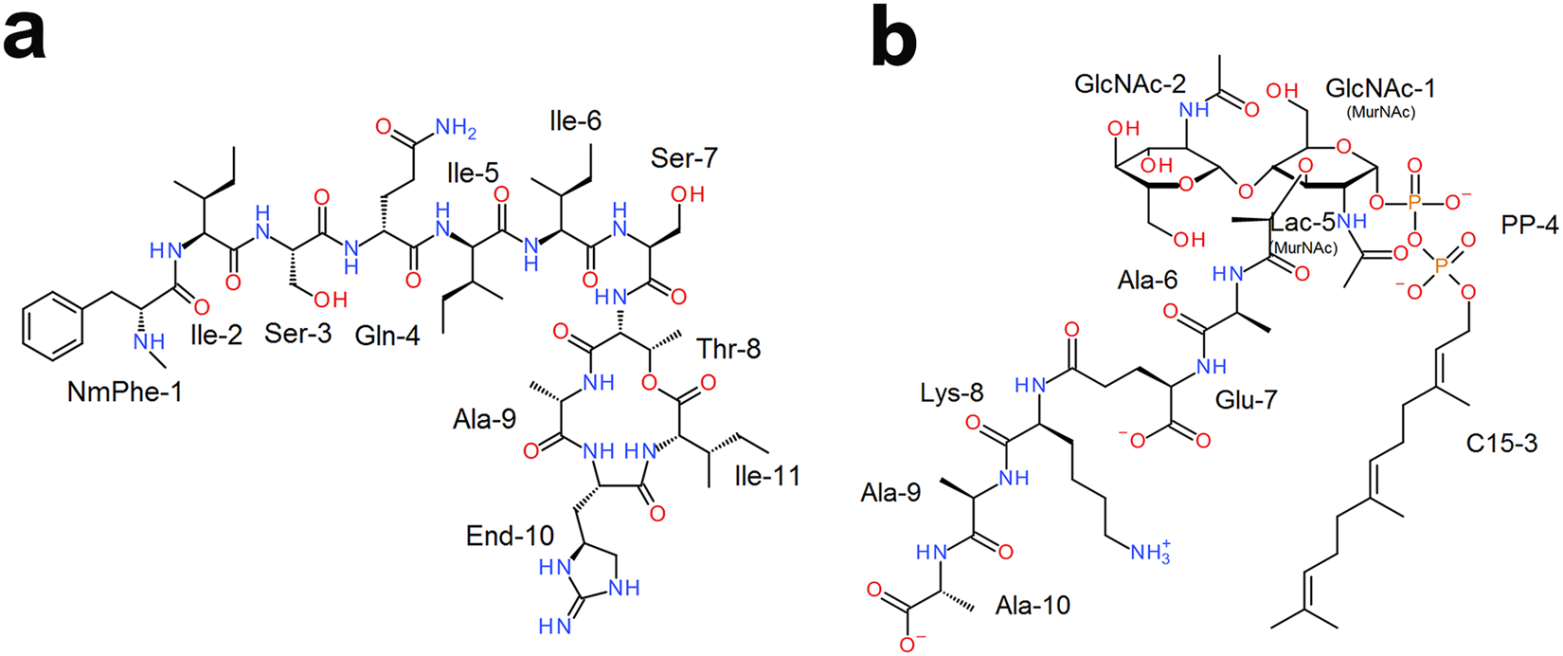
Structure of simulated TXB (**a**) and L_II_ (**b**). TXB contains 11 residues: D-NmPhe-L-Ile-L-Ser-D-Gln-D-Ile-L-Ile-L-Ser-D-Thr-L-Ala-L-End-L-Ile, and the last four residues form a closed ring. (NmPhe: N-methylated phenylalanine, End: enduracididine). L_II_ model used in our simulation was published by Hst *et al*.^17^, which has a shortened hydrocarbon chain (15 carbon atoms) than the wild type (55 carbon atoms).

Recently, Ling *et al*. discovered a new L_II_-targeted antibiotic named teixobactin (TXB) by screening the uncultured bacteria with the help of iChip technology^11^. TXB is produced by the Gram-negative bacterium *Eleftheria terrae* and can effectively kill Gram-positive pathogens without any drug-resistance developed. Structurally, this compound contains 11 amino acids (Fig. 1a), four of which are D-amino acids. Its sequence starts with an N-methylated phenylalanine (NmPhe) and ends with a closed ring, which is formed by the last four residues. TXB forms a complex with L_II_ with a ratio of 2:1 (TXB: L_II_) and interaction of TXB with pyrophosphate and MurNAc group of L_II_ is critical for binding.

The finding of such novel antibiotics stimulated follow-up studies to establish the TXB structure-function relationships. However, the great difficulties in the synthesis of the residue enduracididine (End-10) are the main obstacles in the synthesis of TXB. Jad *et al*. synthesized a TXB analogue by replacing the End-10 residue with an arginine residue^12^, and found this analogue only had a slight increase of the minimum inhibitory concentration (MIC) against Gram-positive bacteria when compared to TXB. Structure-function relationships of TXB and several new analogues indicated that the D-amino acids were important for activity, as almost a total loss of activity was observed when they were replaced by L-amino acids^13,14^. Further, modification of NmPhe-1 by the substitution of a methyl group with an acetyl group^13^, or substitution of the NH proton with one more methyl group, or introduction of a hydroxyl group to aromatic ring^15^ reduced the antimicrobial activity. Nowick’s group reported the importance of the ring motif and suggested the TXB-L_II_ binding was through H bond interaction between amide NH groups on the ring motif of TXB and pyrophosphate group of L_II_^16^, which was similar to nisin-L_II_ binding^17^. The importance of hydrophobicity from the N-terminal tail was also discussed in their work: the TXB analogue still remained bactericidal after replacing the residues NmPhe-1 to Ile-5 with a dodecanoyl group.

These previous studies provided clues on the important function groups of TXB interacting with L_II_. However, a detailed structural model of the TXB-L_II_ complex is not available. In the present work, molecular dynamics (MD) simulations with an enhanced sampling technique were performed to explore the binding phase space of TXB-L_II_ complex with a ratio of 1:1, to figure out the specific binding modes of TXB and L_II_. Four different binding modes are discovered in our simulations, and the results indicate the ring motif of TXB plays an important role in recognizing L_II_. Structural models of the TXB-L_II_ complex with a ratio of 2:1 are suggested, which are also found stable in the lipid bilayer environment. These discoveries provide new insights to understand the binding mechanism of TXB to L_II_.

## Results and Discussion

### Unbiased MD simulations of TXB and L_II_ binding with a ratio of 1:1

**Figure 2:**
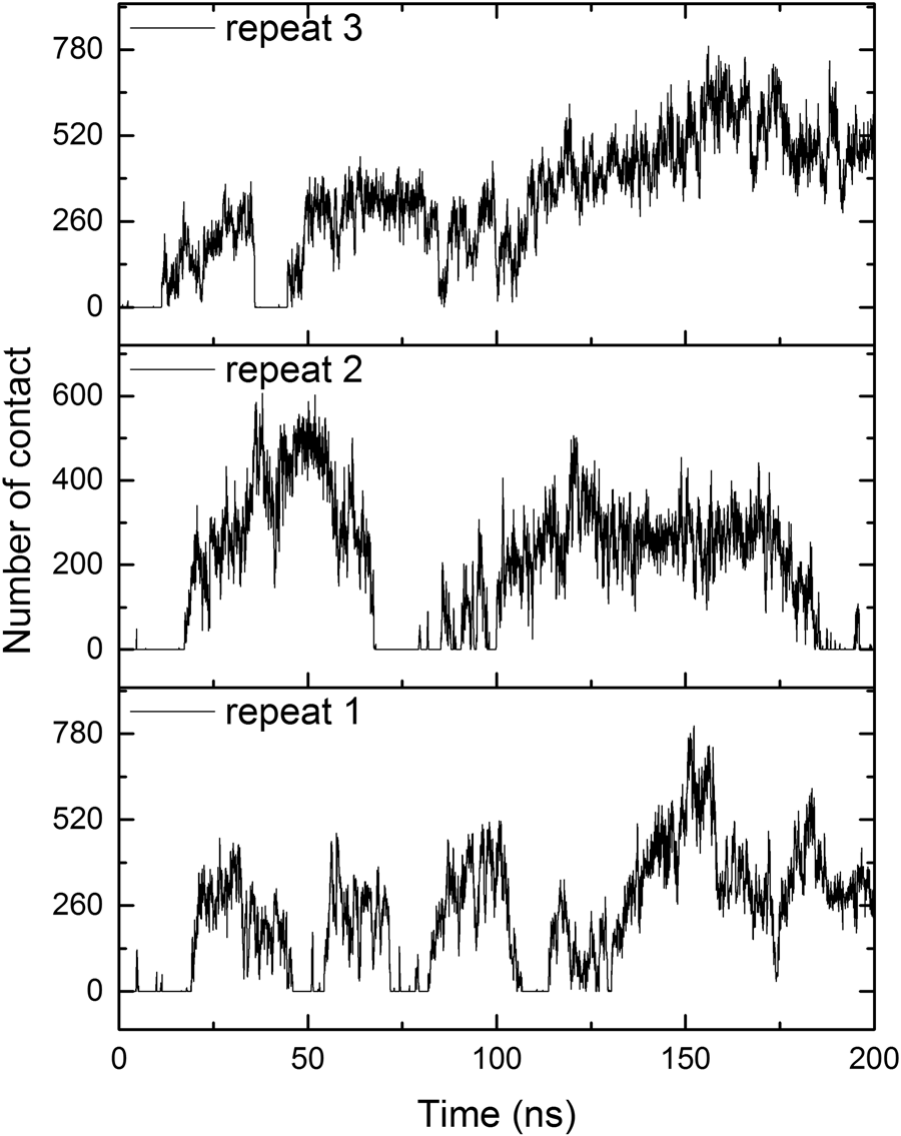
Number of contacts between TXB and L_II_ in three unbiased MD simulation. Only heavy atoms in both molecules were counted. Cutoff was set as 0.6 nm.

The high flexibility for both TXB and L_II_ increases the diversity of binding gestures. With so many polar residues, such as charged residues PP-4, Glu-7, Lys-8, Ala-10 (charged C-terminal residue) in L_II_, and the polar ring motif in TXB, a lot of local minima could be foreseen on the free energy surface (FES) of TXB- L_II_ binding. Three repeats of the simulation were run for 200 ns to investigate the binding of one TXB and one L_II_. The number of contacts between the two molecules (Fig. 2) showed different binding status for each different repeat. However, no stable binding mode could be found from these three repeats and in some cases (Fig. 2 repeat2 & 3) the configuration was trapped in the local minima for about 100 ns. It would be very difficult for an exhaustive exploration by unbiased MD simulations because they are often trapped in the local minima; therefore, an enhanced sampling method is required.

### TXB-L_II_ binding modes explored by PTMetaD-WTE simulation

**Figure 3:**
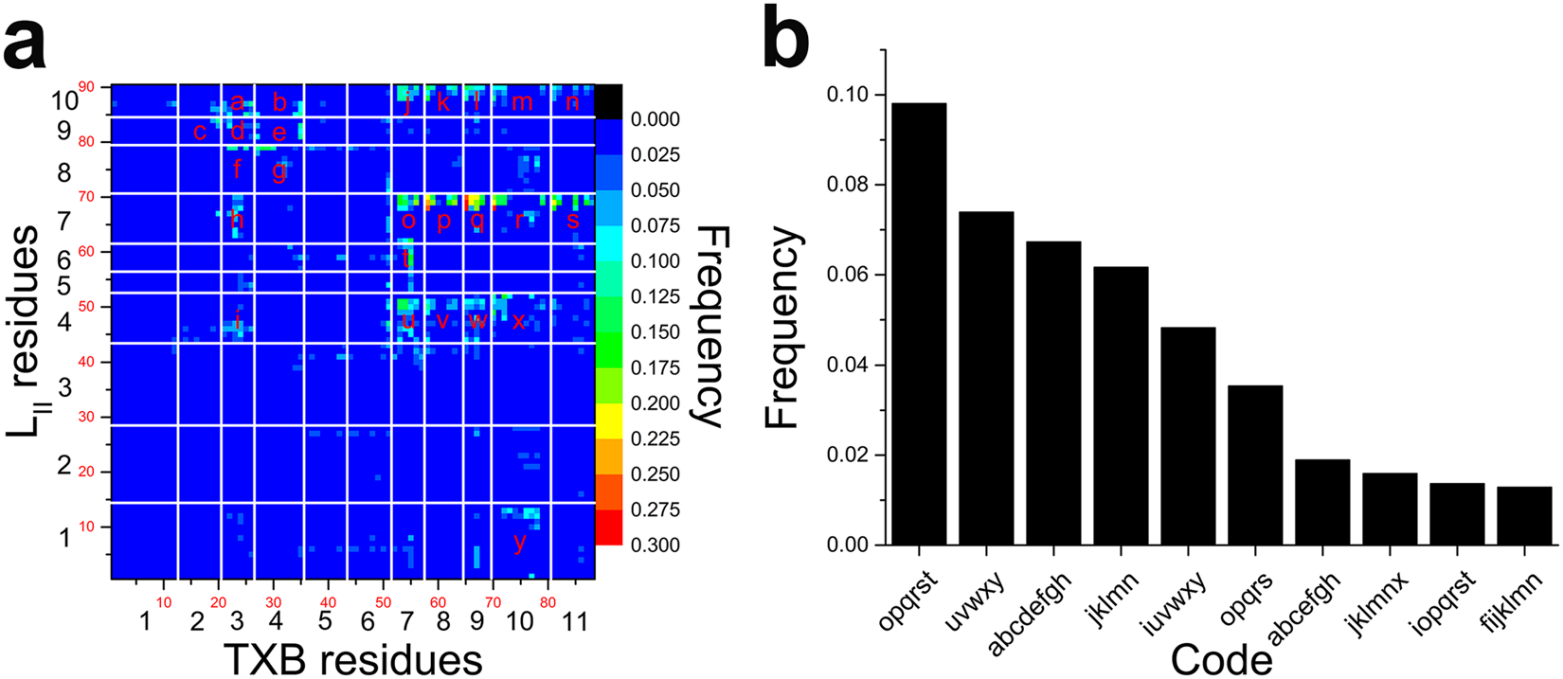
Atom-based contact map (**a**) and histogram of different clusters (**b**). In (**a**) axis labels in red color with smaller size are the heavy atom ID, axis labels in black color with larger size are the residue ID. Based on the contact map, 25 contacted residue-pairs were labelled with the letters from a to y. Each simulated frame was relabeled with these 25 letters by identifying the existing contacts, and clustered into groups. (**b**) Presents the 10 most popular clusters.

The number of contacts in our PTMetaD-WTE simulation of 1 TXB and 1 L_II_ was selected as a CV to describe the system, and each replica was run for 400 ns. Based on the diffusion of the CV and decay of the Gaussian height at 300 K (Fig. S1 in the Supporting Information), as well as the diffusive movement of replicas in the temperature space (Fig. S2), the PTMetaD-WTE simulation was converged. FES of the ensemble at 300 K was constructed by plumed tool, sum_hills (Fig. S3). However, no obvious minima could be found from the one-dimensional FES. Searching for specific binding modes would be difficult for the highly flexible molecules with the structure-based clustering methods. Therefore, the reweighted contact map (Fig. 3a) was utilized to search for specific bindings. The contact map was established based on the heavy-atom pairs of all frames in the low temperature ensemble (300 K). The unbiased weight of each sampled frame was estimated as ${e}^{\beta (V(s(R),t)-c(t))}/Z$ (see Methods), hence the contact-frequency of heavy atoms i and j was calculated as $fre{q}_{i,j}=\frac{{\sum }^{}P{(t)}_{i,j\cdot }{e}^{\beta (V(s(R),t)-c(t))}}{Z}\,$, where *P*(*t*)_*i,j*_ = 1, when the distance between atoms i and j is no larger than 0.4 nm, and *P*(*t*)_*i,j*_ = 0, when their distance is larger than 0.4 nm. Compared with the residue-based contact map, this atom-based contact map avoids the accumulative effects, i.e. several atom pairs with low frequency ends up to one residue pair with high frequency, thus provides contact information with higher accuracy.

According to the contact map, 25 residue-pairs with high occupancy were selected and labelled with the letters from a to y, respectively. The 25 contacts are statistically meaningful, but for a specific binding mode, usually not all the 25 contacts are involved. The next step was to re-label each frame obtained from the simulation by identifying which contacts existed. For example, if a frame is labeled as “opqrst”, it means the contacts of o, p, q, r, s and t exist in this frame. In this way, all the frames could be re-grouped by their labels. The label, which is a combination of the contact letter a to y, functions as a natural metrics for binding mode identification. The 10 most popular binding modes are shown in Fig. 3b (the complete information is presented in Table S1). Among the ten modes, four predominant binding modes were identified, while the other six binding modes in the top 10 list are derivatives of the first 4 modes from adding or missing 1 or 2 contacts.

**Figure 4:**
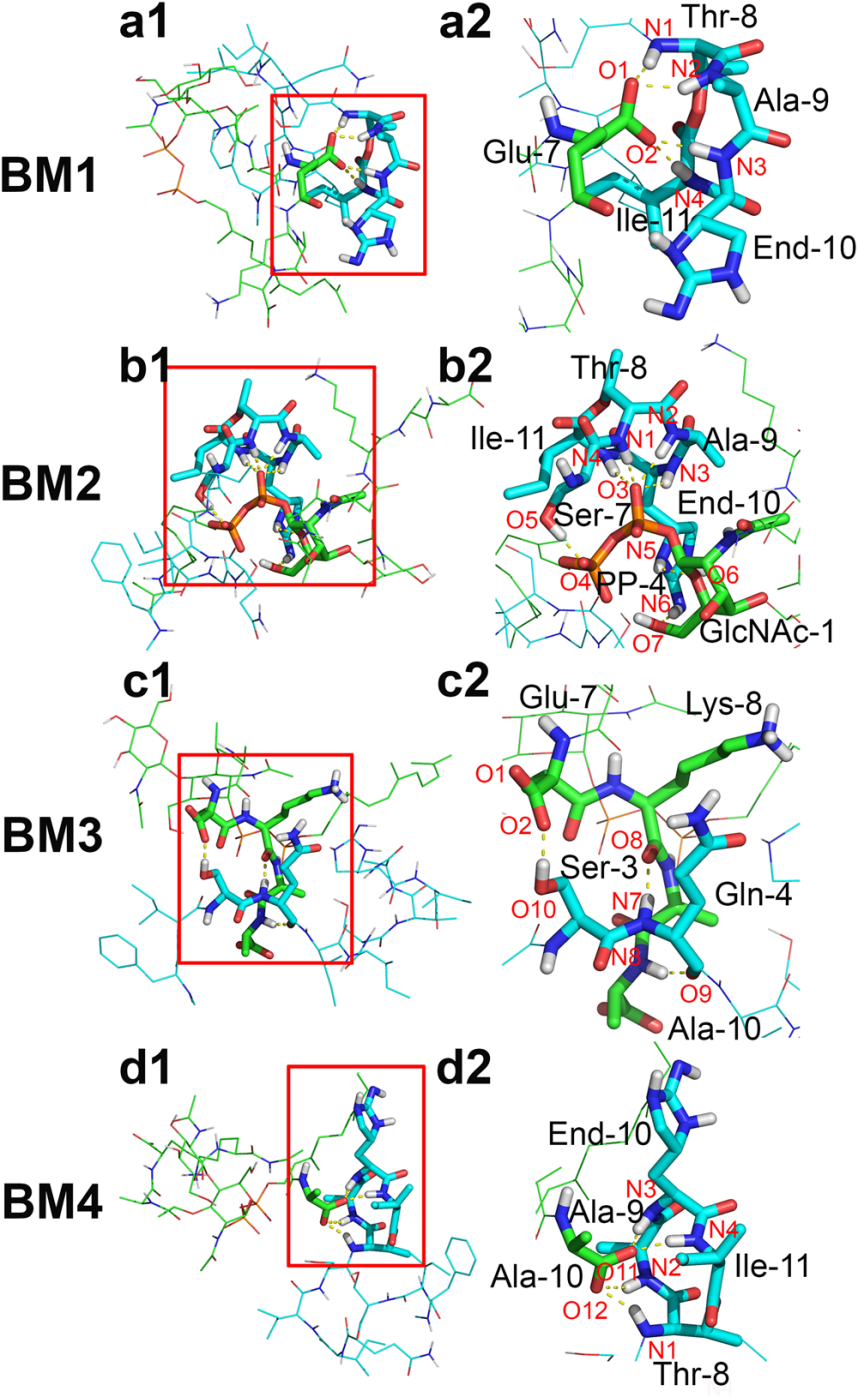
Four binding modes and their featured contacts. In BM1, featured contacts are maintained by four amide groups of the ring motif of TXB form H bonds with Glu-7 of L_II_. In BM2, besides the H bonds between four amide groups of the ring motif of TXB and PP-4 of L_II_, there are also one H bond between Ser-7 of TXB and PP-4 of L_II_, as well as two H bonds between side chain of End-10 of TXB and GlcNAc-1 of L_II_. In BM3, H bonds are formed between Ser-3 of TXB and Glu-7 of L_II_, Gln-4 of TXB and Lys-8 of L_II_, and Gln-4 of TXB and Ala-10 of L_II_. In BM4, H bonds are between ring motif of TXB and Ala-10 of L_II_. Polar hydrogen, nitrogen, oxygen and phosphorus atoms are colored as white, blue, red, and orange respectively, non-polar hydrogen atoms are hidden. Carbon atoms are colored as cyan (TXB) and green (L_II_) to differentiate different molecules. H bonds donor and acceptors are labelled with the red letters.

The gromos^18^ method was used to classify the frames of each binding mode, based on RMSD with respect only to the contacted residues with a cutoff of 0.25 nm. Concretely, that is RMSD of residues Ala-6, Glu-7 (L_II_) and Ser-7, Thr-8, Ala-9, End-10, Ile-11 (TXB) for binding mode 1 (BM1); RMSD of residues GlcNAc-1, PP-4 (L_II_) and Ser-7, Thr-8, Ala-9, End-10 (TXB) for binding mode 2 (BM2); RMSD of residues Glu-7, Lys-8, Ala-9, Ala-10 (L_II_) and Ile-2, Ser-3, Gln-4 (TXB) for binding mode 3 (BM3); and RMSD of residues Ala-10 (L_II_) and Ser-7, Thr-8, Ala-9, End-10, Ile-11 (TXB) for binding mode 4 (BM4). The representative conformations of each binding mode are presented in Fig. 4a1,b1,c1 and d1, and their featured contacts are in Fig. 4a2,b2,c2 and d2, respectively. Starting from these representative structures, three repeats of standard MD simulation, each of which had a length of 200 ns, were performed for each binding mode. Stabilities of these binding modes were confirmed by comparing the contact maps for the first 10 ns simulation and last 10 ns simulation of each MD repeat (Fig. S4). In all four cases, both molecules in the complex remain very flexible (in the end of the simulations, the whole molecule RMSDs could reach 1.0 nm, 0.9 nm, 0.4 nm, and 0.8 nm for BM1, BM2, BM3 and BM4 systems, respectively. The RMSDs are shown in Fig. S5) and more importantly, the featured contacts between them were maintained throughout the whole simulations (the featured contacting residues RMSDs could only reach 0.15 nm, 0.15 nm, 0.2 nm, and 0.15 nm for BM1, BM2, BM3 and BM4 systems, respectively Fig. S5).

The featured contacts of BM1 were mainly maintained by the H bonds interaction between the four amide groups on the TXB ring motif (from residue Thr-8 to residue Ile-11) and the carboxyl group of Glu-7 on L_II_. BM2 was characterized by the H bonds between four amide groups on TXB ring motif and PP-4 of L_II_, additionally, one H bond was also found between the hydroxyl group of Ser-7 of TXB and PP-4 of L_II_, as well as two H bonds between the sidechain-ring of End-10 of TXB and GlcNAc-1 of L_II_. BM3 was stabilized by three H bonds one of which is formed between the backbone carbonyl group of Gln-4 and the amide group of Ala-10, one is between the backbone amide group of Gln-4 and the carbonyl group of Lys-8, and another one is formed between the hydroxyl group of Ser-3 and the carboxyl group of Glu-7. And BM4 was similar to BM1, the difference is the carboxyl group was from Ala-10 instead of Glu-7. Occupancy for each of these H bonds during 600 ns simulation (3 repeats with 200 ns each) is listed in Table 1.

**Table 1:** Details of H bonds maintaining the featured contacts in each binding mode.

Binding Mode	Donor	Accepter	Occupancy (%)
BM1	N1H (Thr-8)	O1 (Glu-7)	63.0
	N2H (Ala-9)	O1 (Glu-7)	52.8
	N3H (End-10)	O1 (Glu-7)	50.1
	N4H (Ile-11)	O1 (Glu-7)	45.3
	N1H (Thr-8)	O2 (Glu-7)	69.9
	N2H (Ala-9)	O2 (Glu-7)	47.6
	N3H (End-10)	O2 (Glu-7)	47.3
	N4H (Ile-11)	O2 (Glu-7)	51.0
BM2	N1H (Thr-8)	O3 (PP-4)	99.4
	N2H (Ala-9)	O3 (PP-4)	81.5
	N3H (End-10)	O3 (PP-4)	70.7
	N4H (Ile-11)	O3 (PP-4)	83.8
	O5H (Ser-7)	O4 (PP-4)	90.5
	N5H (End-10)	O6 (GlcNAc-1)	57.5
	N6H (End-10)	O7 (GlcNAc-1)	51.5
BM3	O10H (Ser-3)	O1 (Glu-7)	41.2
	O10H (Ser-3)	O2 (Glu-7)	34.5
	N7H (Gln-4)	O8 (Lys-8)	98.2
	N8H (Ala-10)	O9 (Gln-4)	81.3
BM4	N1H (Thr-8)	O11 (Ala-10)	75.4
	N2H (Ala-9)	O11 (Ala-10)	66.4
	N3H (End-10)	O11 (Ala-10)	49.1
	N4H (Ile-11)	O11 (Ala-10)	58.8
	N1H (Thr-8)	O12 (Ala-10)	40.9
	N2H (Ala-9)	O12 (Ala-10)	34.6
	N3H (End-10)	O12 (Ala-10)	42.2
	N4H (Ile-11)	O12 (Ala-10)	24.7

The occupancy was calculated over three repeats of MD simulation with 200 ns each. Atom names could be found in Fig. 4.

Further analysis of the featured contacts of BM1 and BM4 finds that the carboxyl groups of Glu-7 and Ala-10 come from the backbone: Glu-7 covalently bonds to Lys-8 via the side chain carboxylate instead of the backbone carboxyl and Ala-10 is located at the C-terminus. These two carboxyl groups cannot be changed by side-chain mutations. Both carboxyl groups could rotate during the binding. Therefore, both oxygen atoms in each carboxyl have the opportunity to form H bonds with the four amide groups in the ring motif of TXB (Table 1). On average, BM1 has 4.27 H bonds (obtained by simply adding the occupancy) between the carboxyl and the ring motif, while BM4 has 3.92. Thus, we suggest that BM1 is slightly more stable than BM4.

In BM2, the phosphate groups of L_II_ interact with the ring motif and Ser-7 of TXB individually (Fig. 4b2). They both have H bonds with occupancy higher than 90% (Table 1), indicating the interaction is very strong. The sidechain of End-10 of TXB also forms two H bonds with GlcNAc-1 of L_II_ to further strengthen this binding. On average, the total number of H bonds in BM2 could reach 5.35, which is higher than BM1 and BM4.

**Figure 5:**
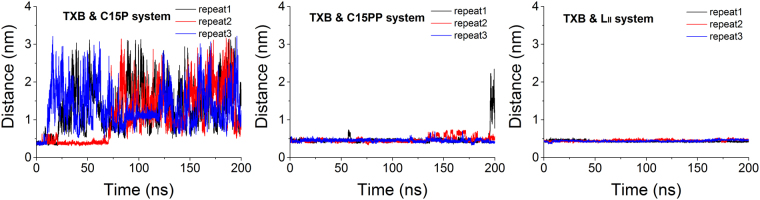
Distance between ring motif of TXB and phosphate group (C15P, left panel) or pyrophosphate group (C15PP and L_II_, middle and right panel, respectively). Binding in TXB & L_II_ system is the most stable out of three, indicating that both pyrophosphate moiety and the first sugar moiety of L_II_ are required for a stable binding with ring motif of TXB.

Since the ring motif of TXB makes polar-contacts only with one phosphate group of the pyrophosphate residue, we examined whether one phosphate group is enough for a stable binding. Two new simulation systems were designed to verify the importance of the PP-4 motif. One system contained one TXB molecule and one C15P molecule, which was composed of a bactoprenol carbon chain covalently bonded to a phosphate group. The other system contained one TXB and one C15PP molecule, which was made of a carbon chain and a pyrophosphate group. Force field parameters for both C15P and C15PP molecules were taken from GAFF. Each system was run by triplicate and simulations started from the bound conformation. Distances between the ring motif and the phosphate (TXB & C15P system) or the pyrophosphate (TXB & C15PP system and TXB & L_II_ system) are shown in Fig. 5. Data showed that binding of the ring motif and the phosphate (TXB & C15P system) was unstable. Adding one more phosphate to the system (TXB & C15PP system) rendered binding much more stable because of the higher polarity of the pyrophosphate group than one phosphate group. However, binding in TXB & C15PP system was not as stable as that in TXB & L_II_ system. The inter-group distance in TXB-C15PP system showed larger fluctuation than that in TXB-L_II_ system at the end of simulations, and unbinding happened in repeat 1 of TXB-C15PP system, indicating that the introduction of GlcNAc-1 in L_II_ further stabilized the binding. Our results agree with the experimental finding^11^ that the minimal motif of L_II_ required for stable binding with TXB is the pyrophosphate (PP-4) and the first sugar (GlcNAc-1) moieties, both of which are highly conserved in bacteria^2^.

**Figure 6:**
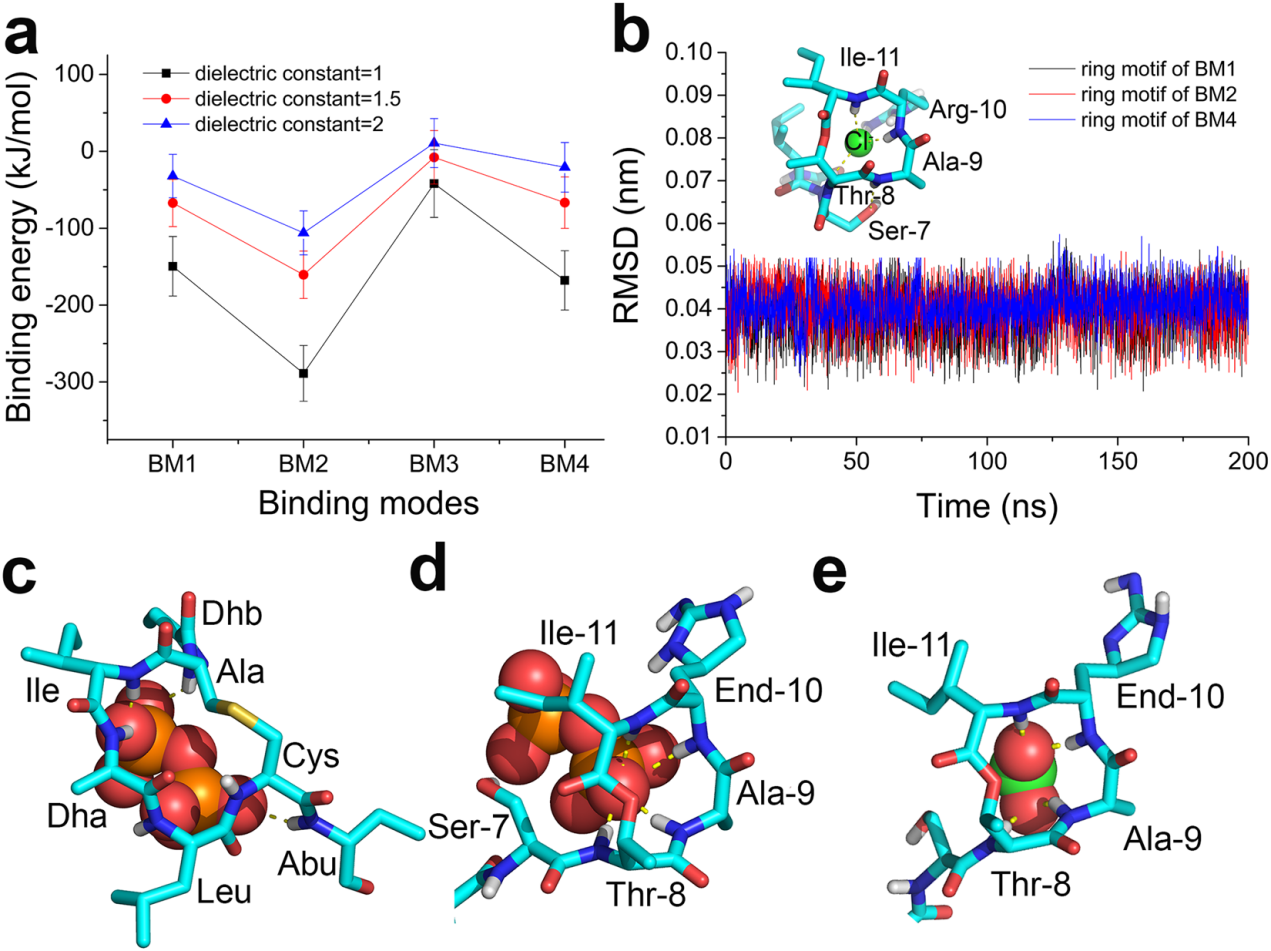
Binding energy of different binding modes (**a**). 100 conformations from last 5 ns of the stability-check simulation of each binding mode were used for the calculation. Binding affinity in a descending order is BM2 > BM1 > BM4 > BM3, indicating the importance of ring motif. We superimposed the ring motifs (BM1, BM2, and BM4) onto the ring motif of a published x-ray crystallographic structure (**b** embedded figure), and calculated RMSD of our ring motif with respect to the published ring motif (**b**). Side chains were not included in the RMSD calculation, since the published structure was a TXB analogue, which has an Arg-10 residue rather than End-10 residue. The fundamental interactions in these ring-involved binding modes share features with those in nisin-L_II_ binding: in nisin-L_II_ binding, amide groups on the ring backbone H bond with negatively charged pyrophosphate (**c**); similarly, in TXB-L_II_ binding, amide groups on the ring backbone also form H bonds with negatively charged pyrophosphate (BM2, **d**), or with negatively charged carboxyl (BM1/BM4, **e**). Negatively charged groups are shown in sphere models: pyrophosphate groups in (**c**,**d**), and carboxyl group in (**e**).

The binding energies of the four binding modes were estimated (Fig. 6a) using the MM-PBSA method with the tool of g_mmpbsa published by Kumari *et al*.^19^. However, in MM-PBSA method^20^, selection of the dielectric constant of the solute would be tricky^21,22^. The charge-charge interaction related terms in binding energy, such as the polar solvation term, and especially the electrostatics term, can be largely affected by the solute dielectric constant. We adopted three different dielectric constants, and although the binding energy of each binding mode varied at different dielectric constants, the overall trend at the same dielectric constant is that E_BM2_ < E_BM1_ < E_BM4_ < E_BM3_, suggesting that BM2 has the highest binding affinity among the four.

Three out of the four binding modes (BM1, BM2 and BM4) utilize the ring motif of TXB to attract the charged residues of L_II_, indicating that TXB ring motif plays a critical role in recognizing L_II_. Recently, a series of TXB analogues with varying structure and stereochemistry were synthesized to elucidate the TXB pharmacophore. The ring structure was found critical to bactericide function^16^. Further, a crystallographic structure of a TXB analogue binding with a chloride anion^23^ shows the H bonding between the ring motif and a negatively charged ion (Fig. 6b embedded). To validate our predicted ring structure, the root mean squared deviations (RMSD) of the backbone atoms of the ring motif with respective to the crystallographic structure were calculated (Fig. 6b). The RMSD values were only around 0.4 Å for BM1, BM2 and BM4, indicating our predicted ring structure agrees with the crystalized structure. The published structure also supported the H bonding pattern predicted by our model. However, our model has some differences with the crystallographic structure where the amide group of Ala-9 (TXB) is H bonded with the hydroxyl group of Ser-7 (TXB), and the other three amide groups interact with the chloride anion. In the ring motif of our model, all four amide groups on the backbone form H bonds with the negatively charged residues.

Comparisons of the binding modes of TXB with other L_II_ binders show some differences and similarities. For instance, vancomycin binds to the negatively charged residue (the D-Ala-D-Ala segment of L_II_)^6,7^ through the formation of a “carboxylate anion binding pocket”^24^. Tridecaptin A1, a Gram-negative L_II_ binder (Gram-negative L_II_ has a meso-diaminopimelic acid instead of a lysine on the penta-peptide motif), forms H bonds with the carboxylate of meso-diaminopimelic acid, and the pyrophosphate moiety is not involved in the binding^25^. Mersacidin has an electrostatic-interaction dominated binding to L_II_, and the terminal GlcNAc sugar is included in the binding^26^. In contrast, Nisin binds to L_II_ through the H bonds between the backbone amides on the ring structure and the pyrophosphate moiety^17^, and shares features with TXB (Fig. 6c–e). We performed 100 ns unbiased MD simulation of nisin-L_II_ complex starting from the published structure (pdb: 1WCO) to test the reliability of our force field parameters. The topology file of nisin was developed with the same strategy as we described in Methods. The nisin-L_II_ binding mode was well maintained during the 100 ns (Fig. S6), indicating that our force field parameters are reliable for the binding study.

### Prediction of TXB-L_II_ complex with a ratio of 2:1

Experiments found that one L_II_ could form stable complex with two TXB molecules^11^. Based on the four 1:1 binding modes found in this study, the structural models of six different 2:1 complexes, labelled as BM1&BM2, BM1&BM3, BM1&BM4, BM2&BM3, BM2&BM4, and BM3&BM4 were constructed and validated through MD simulations with three repeats each for a length of 200 ns.

**Figure 7:**
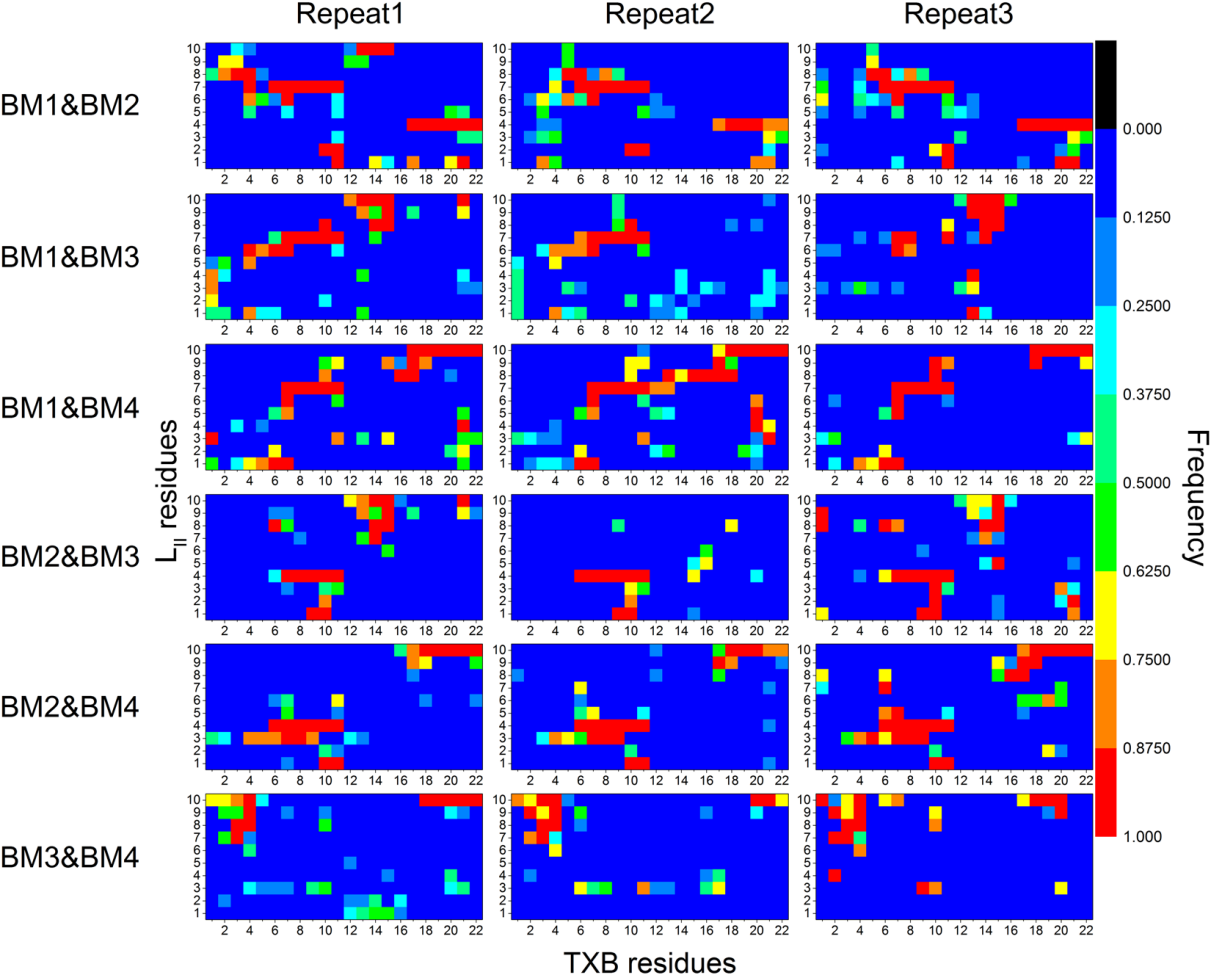
Contact map of different TXB-L_II_ complex with a ratio of 2:1. Each complex was run for 3 repeats with 200 ns each, and the last 50 ns trajectory was used for the calculation of contact map. Only heavy atoms were considered, and the contact cutoff was set as 0.4 nm. Residue number from 1 to 11 on x axis belonged to one TXB, and from 12 to 22 belonged to another TXB. Featured contacts areas are highlighted with ellipses.

The contact map of the last 50 ns is presented in Fig. 7, and all the featured contacts region were highlighted with the ellipses. In both BM1&BM3 and BM2&BM3, the featured contacts for BM3 were not found in one repeat, and in BM3&BM4, the featured contacts of BM4 partially disappeared, indicating that all these three 2:1 models were not as stable as the BM1&BM2, BM1&BM4 or BM2&BM4. Our results suggest that in TXB-L_II_ binding, two ring motifs of TXB could bind to any two out of the three binding sites supplied by L_II_ (PP-4 & GlcNAc-1 moiety, Glu-7 and Ala-10). However, considering the relatively higher binding affinity of BM1 and BM2, we believe that the model BM1&BM2 is the most probable model.

Another possibility for the 2:1 binding mode is that TXB molecules first form a dimer, and then bind to one L_II_ molecule. Such binding mode was found for ramoplanin, which dimerizes under membrane environment before binding to L_II_^27^. Therefore, another 400 ns PTMetaD-WTE simulation was carried out to simulate the interaction between two TXB molecules in solution. Based on the contact map and the subsequent unbiased MD simulation (Figs S7, S8), a TXB dimer with a featured-contact between the ring motif of one TXB molecule and the residue Ile-6 of another TXB molecule was found stable. A possible 2:1 TXB-L_II_ model was created by positioning a L_II_ molecule close to the other ring motif of the TXB dimer with the pyrophosphate group based on the binding mode of BM2. Nevertheless, this complex was not stable: the ring motif which bound to the Ile-6 promptly lost contacts while the other ring motif strongly attached to the pyrophosphate group of L_II_ (Fig. S9). These results suggest that TXB molecules could dimerise in solution, but this dimer is not stable under the competition of L_II_.

**Figure 8:**
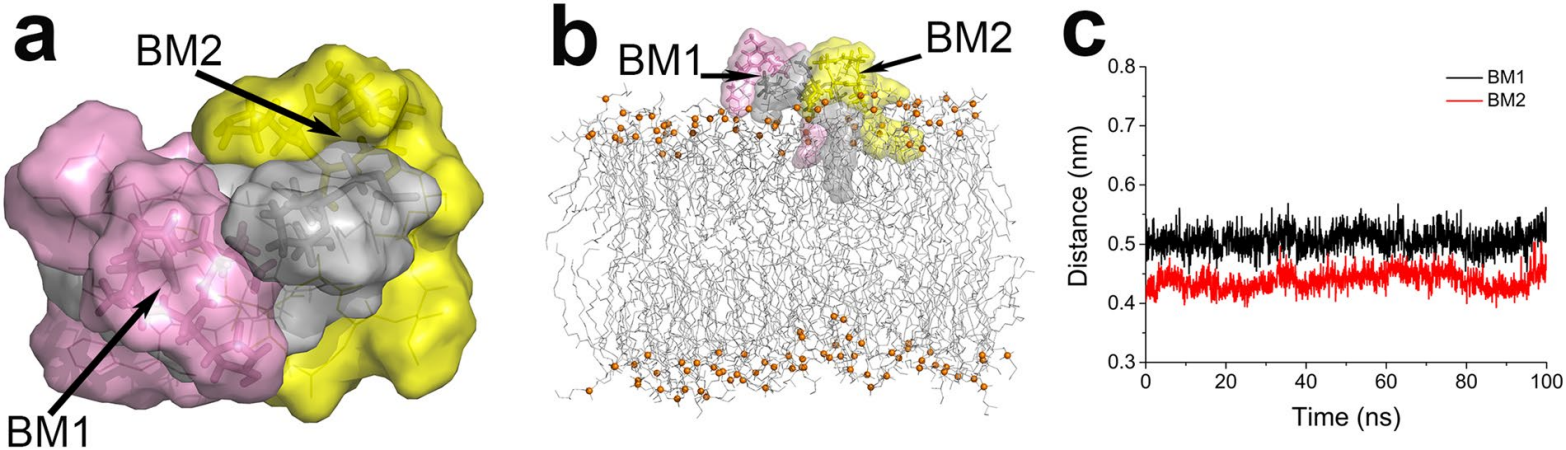
Surface model of stable TXB-L_II_ complex with a ratio of 2:1 (**a**). This complex was built based on featured contacts of BM1 and BM2, e.g., the ring motifs of two TXB molecules bound to the PP-4 & GlcNAc-1 moiety and Glu-7 residue of a same L_II_. Stable conformation of 2:1 complex in membrane environment (**b**). Distances between the first ring motif and Glu-7 (BM1) and the distance between the second ring motif and PP-4 (BM2) during the simulation in membrane environment (**c**). Two TXB molecules are colored as pink and yellow, respectively, and L_II_ molecule is colored as gray. Lipid molecules in membrane model are colored as gray lines, while their phosphorus atoms are shown as orange sphere to state the position of their head groups.

Therefore, we favour the structural model of 2:1 TXB-L_II_ complex (Fig. 8a), in which the ring motif of two TXB molecules bound to the PP-4 & GlcNAc-1 moiety and Glu-7 residue of the same L_II_ respectively. These two contacts function as anchors, while the rest of TXB can move freely since no specific binding patterns were found between the rest residues of both TXB and L_II_ (Fig. 7 BM1&BM2) or between the two TXB molecules (Fig. S10). Further, this complex was simulated under a membrane environment to verify its stability (Fig. 8b). The distances between the ring motif of the first TXB and Glu-7 and the distance between the ring motif of the second TXB and PP-4 were monitored (Fig. 8c). The complex was stable in 100 ns MD simulation.

## Conclusion

Using PTMetaD-WTE simulations, we explored the binding phase space between the two molecules of TXB and L_II_. We also developed a strategy to accurately cluster structures for a system with very high flexibility, which was realized by labeling each structure with the featured residue-contacts and grouping the structures with these labels. Four binding modes (BM1, BM2, BM3 and BM4) were discovered. These structural models explain several experimental observations by confirming that: 1) the pyrophosphate-MurNAc moiety of L_II_ is the minimal motif for a stable binding in BM1; 2) The ring motif of TXB is critical for its bactericide function; and 3) Binding sites in L_II_, the pyrophosphate-MurNAc moiety, the carboxyl group on the backbone of glutamic acid residue and the carboxyl group on the backbone of C-terminus alanine residue play essential roles in the synthesis of the bacterial cell wall. Thus, TXB resistance mutants of L_II_ are unlikely to develop. To further validate such binding modes, X-ray and NMR approaches may be needed. Based on the flexible nature of such binding it is anticipated that direct structure characterization with the crystallography or NMR may be difficult. With our predicted complex models as a working hypothesis, experiments can be designed to explore the recognition mechanism between TXB and L_II_.

## Methods

### Unbiased MD simulation details

All the amino acid residues in TXB and L_II_ molecules are described by AMBER99SB force field^28^, except End-10 in TXB, which is not defined in AMBER99SB. We chose GLYCAM_06j-1 force field^29^ to describe the sugar residues GlcNAc-2 in L_II_. However, the other sugar residue MurNAc was not available in the force field. MurNAc could be considered as the ether of lactic acid and GlcNAc, so it was divided into two residues GlcNAc-1 and Lac-5. Same as End-10 in TXB, Lac-5 is not a standard residue either, therefore, we generated their topologies, as well as the topologies of other undefined residues like C15-3 and PP-4 in L_II_, based on generalized amber force field (GAFF)^30^. Partial charges for the undefined residues (End-10 of TXB, C15-3, PP-4 and Lac-5 of L_II_) were fitted based on Restrained Electrostatic Potential (RESP) method in which the electric potential was calculated with Gaussian 09 at the level of HF/6-31 G*. The partial charges of some residues on the ring motif of TXB and Glu-7 of L_II_ bonded with the neighboring residues in a non-standard way were recalculated using the same strategy.

MD simulations were performed with GROMACS package, version 4.6.7^31^. We mainly focused on the interaction between TXB and L_II_; therefore, the membrane was not included in the exploration of the binding phase space of TXB-L_II_ complex to save the computational resources. This is reasonable according to the original study^11^, which claimed that purified L_II_ could form complex with TXB *in vitro*, indicating the membrane is not involved in TXB-L_II_ binding. The simulation systems were composed of TXB, L_II_ (ratio of them would be mentioned in results and discussion part), TIP3P water^32^, and counter ions NaCl. Counter ions were modified by Joung *et al*.^33^ and added to a concentration of 0.17 M while neutralizing charges of the simulation systems. A leap-frog algorithm was used to integrate Newton’s equation of motion, and the time step was set to 2 fs along with the application of LINCS algorithm^34^, by whom all the covalent bonds between hydrogen atoms and heavy atoms were constrained. The cutoff for both van der Walls (VDW) interaction and short-range electrostatic interaction were set to 1.0 nm, and a Particle mesh Ewald (PME)^35^ method was employed to deal with the long-range electrostatic interaction. After the energy minimization, two short simulations with position restrains of all atoms of TXB and L_II_ were performed to equilibrate the simulation system: 100 ps simulation in NVT ensemble to heat up the system temperature to 300 K and another 100 ps simulation in NPT ensemble to adjust the system pressure to 1 bar. After that, the product simulations were run 200 ns in NPT ensemble with all position restrains removed. The temperature and pressure of the system were maintained by V-rescale temperature coupling method and Parrinello-Rahman pressure coupling method respectively. Each kind of simulation was repeated three times by random assigning initial velocities.

After the TXB-L_II_ complex was stabilized in the aqueous simulations, we checked the stability of the complex in a membrane environment. The membrane model was built utilizing a web server MemBuilder II and parameterized with a Slipids force field published by Jambeck and Lyubertsev^36–38^, which is compatible with AMBER force field. In the prepared lipid bilayer, each leaflet of the membrane contained 48 negatively charged POPG and 16 zwitterionic POPE. Then one POPG was removed from the upper leaflet and our lipid II model was embedded. The membrane model was equilibrated for 50 ns at 300 K and 1 bar, and its area per lipid (APL) reached a value of 0.60 nm^2^, which was comparable with experimental results (0.59 nm^2^ for pure POPE at 303K^39^, and 0.64 nm^2^ for pure POPG at 293K^40^). After that, two TXB molecules were added onto the L_II_ molecule in the predicted 2:1 complex configuration in the simulation box and 100 ns production MD simulation was performed. The distances between the ring motifs of the two TXBs and their respective targeting residues of L_II_ were measured to illustrate the stability of the TXB-L_II_ complex.

### Parallel tempering metadynamics simulation in well- tempered ensemble (PTMetaD-WTE)

In most cases, MD simulation is useful in exploring interesting events only when the system is ergodic in the simulated timescale. However, practically, simulation system usually presents metastability, and sampling would be trapped by some local minima of free-energy, making sufficient sampling unaffordable. Therefore, it is always a challenge for MD simulation to efficiently explore the large regions of phase space due to the limited time scale that the computational resources could reach.

Metadynamics^41,42^ is one of the most popular sampling-enhancing methods, especially to validate and efficiently probe rare events, and to reconstruct free energy surfaces (FES)^43–46^. In metadynamics, a small repulsive Gaussian shaped potential, which is the function of the selected collective variables (CVs), is added to internal potential of the system every specified number of MD steps. At time t, the accumulated Gaussian potential *V*_G_(*s,t*) can be written as,

1 $${V}_{G}(s,t)={\int }_{0}^{t}dt^{\prime} \omega \,\exp (-\sum _{i=1}^{d}\frac{{({s}_{i}(R)-{s}_{i}(R(t^{\prime} )))}^{2}}{2{\sigma }_{i}^{2}})\,$$

where *R* is the microscopic coordinates and $s(R)=({s}_{1}(R),\ldots ,{s}_{d}(R))$, a set of selected CV, is the function of *R*. σ_*i*_ is the Gaussian width and $\omega$ is an energy rate, which could be expressed as the ratio of Gaussian height *W* and deposition stride *τ*_G_: $\omega =W/{\tau }_{G}$. Impact of this external bias potential is to disfavor the more frequently visited configurations. In this way, sufficient statistics for every CV value could be collected and FES could be reconstructed along the CV space.

Barducci *et al*. published a well-tempered metadynamics in 2008^47^ to improve the convergence of metadynamics by utilizing a modified external bias,

2 $$V(s,t)={\rm{\Delta }}Tln(1+\frac{\omega N(s,t)}{{\rm{\Delta }}T})$$

where $N(s,t)={\int }_{0}^{t}{\delta }_{s,s(t^{\prime} )}dt^{\prime}$, is the histogram of CV and its time derivative $\dot{N(s,t)}={\delta }_{s,s(t)}$. Δ*T* has a temperature dimension.

Time derivative of this bias potential could easily be obtained,

3 $$V(\dot{s},t)=\frac{\omega {\rm{\Delta }}T{\delta }_{s,s(t)}}{{\rm{\Delta }}T+\omega N(s,t)}=\omega {e}^{-V(s,t)/{\rm{\Delta }}T}{\delta }_{s,s(t)}$$

and if the *δ*_*s*_,_*s*(*t*)_ is replaced by Gaussian, the difference between equation (3) and time derivative of equation (1) would only lies in the factor ${e}^{-V(s,t)/{\rm{\Delta }}T}$, which is usually integrated into Gaussian height in practice: $W=\omega {e}^{-V(s,t)/{\rm{\Delta }}T}{\tau }_{G}$. By using the new bias, Gaussian deposition rate would decrease over simulation time, leading to the convergence of *V*(*s*,*t*) to underlying free energy rather than oscillating around it and greatly increasing the availability of metadynamics. However, some hidden degrees of freedom that could not be described by the chosen CVs, would still limit exploration of the phase space, especially for those systems with high conformational complexity.

Another commonly used method to enhance sampling is Replica Exchange MD (REMD), such as parallel tempering (PT). In PT, non-interacting replicas of the system are simulated with different temperatures and exchange with adjacent replicas. Energy barriers might be overcome when higher temperatures are accessed by replicas, allowing for the exploration of new conformational space. Metadynamics can be easily combined with PT (PTmetaD), and will dramatically enhance the sampling efficiency of each replica. Although PTmetaD compensates the weakness of PT or Metadynamics in some degree, a sufficient overlap between the potential energy distributions of neighboring temperatures is necessary to reach an efficient exchange, which usually means the temperature gap cannot be too large and lots of replicas are required to achieve the sampling at a high temperature. However, in well-tempered ensemble (WTE)^48^, which is realized by well-tempered metadynamics when the system energy is used as CV, fluctuations of energy are enlarged, making it possible to have an efficient exchange with less replicas. Sampling efficiency of PTMetaD-WTE was used for protein folding^49,50^, peptide adsorption onto surfaces^51^, and protein oligomerization^52^.

Our PTMetaD-WTE simulation was performed using Gromacs 4.6.7 package with the help of plumed 2.1.3 plug-in^53^. 16 replicas were simulated in NVT ensemble to avoid the instability of simulation box in NPT ensemble at high temperature. Temperatures varied from 300 K to 546.6 K and followed an exponential distribution: ${T}_{i}={T}_{0}\ast {e}^{k\ast i}$, where *T*_*i*_ was the temperature of replica i, and k = 0.04. Each replica contained 1 TXB, 1 L_II_, 6506 TIP3P water molecules as well as 24 sodium ions and 21 chlorine ions. 16 starting conformations of the TXB-LII complex with different contacts were generated from a standard MD simulation at high temperature (500 K). Before the PTMetaD-WTE simulations, 500 ps simulation was performed to equilibrate each replica.

PTMetaD-WTE simulations were separated into two steps. First, 20 ns PTMetaD simulation was run with biases only on the potential energy. During this step, Gaussian potential was added every 500 calculation steps (1 ps), and a bias factor equal to 60 was adopted. Gaussian height was set to 2 kJ/mol and Gaussian width was set to 500 kJ/mol. Probabilities of neighboring-replica-exchange could reach 45%. In the second step, the accumulated biases on the potential energy CV in the first step were kept as a static additional bias potential, but no more bias were added to the potential energy. Number of contacts between TXB and L_II_ heavy atoms was used as the new biased CV. In metadynamics simulation, CV should be wisely chosen to describe the degrees of freedom of system to achieve convergence. However, with the benefit from WTE, such limitation is largely reduced. The reason we chose the number of contacts as CV, instead of other generic CV such as inter-molecular distance, is that the contacts naturally include the information of interacting pairs between the two molecules, which is closely related to the binding modes. Number of contacts $S$ was defined as the number of heavy atom pairs with a distance smaller than 0.6 nm, within which each heavy atom was from different molecules. Its value became differentiable by adopting a switching function:

4 $$S=\sum _{i\in A}\sum _{j\in B}\frac{1-{(\frac{{r}_{ij}}{{r}_{0}})}^{6}}{1-{(\frac{{r}_{ij}}{{r}_{0}})}^{12}}$$

where *i* and *j* were labels of heavy atoms from molecule A and B respectively, ${r}_{ij}$ was the pair distance and ${r}_{0}$ (0.6 nm) was the distance cutoff. During this step, values of bias factor and Gaussian width were set as 8 and 5 respectively, Gaussian height and time interval of Gaussian deposition were still 2 kJ/mol and 1 ps. 400 ns PTMetaD-WTE simulation was finished for each replica.

In metadynamics simulation, weight of each sampled frame is biased by the additional Gaussians. The unbiased weight of each frame can be obtained from biased one based on the script published along with this work^54^. The instantaneous biased frequency $P(R,t)$ has an expression as:

5 $$P(R,t)=\frac{{e}^{-\beta (U(R)+V(s(R),t))}}{{\int }^{}dR{e}^{-\beta (U(R)+V(s(R),t))}}$$

where *U* (*R*) is internal potential, and *β* is the inverse of product of temperature and Boltzmann constant. Equation (5) can be deformed into^55^:

6 $$P(R,t)={e}^{-\beta (V(s(R),t)-c(t))}\cdot {P}_{0}(R)$$

where ${P}_{0}(R)=\frac{{e}^{-\beta U(R)}}{{\int }^{}dR{e}^{-\beta U(R)}}$ is the unbiased Boltzmann distribution, and c(t) has a form as:

7 $$c(t)=\frac{1}{\beta }ln\frac{{\int }^{}ds\,{e}^{-\beta F(s)}}{{\int }^{}ds{e}^{-\beta (F(s)+V(s,t))}}$$

where *F*(*s*) is the free energy as a function of CV $s$.

Therefore, the unbiased weight of each sampled frame has a value of ${e}^{\beta (V(s(R),t)-c(t))}/Z$, where $Z={\int }^{}dt{e}^{\beta (V(s(R),t)-c(t))}$ is a constant.

### Data Availability Statement

The datasets generated during and/or analysed during the current study are available from the corresponding author on reasonable request.

## Electronic supplementary material

Supplementary information
