# Supplementary material for: Binding Modes of Teixobactin to Lipid II: Molecular Dynamics Study
Source: Sci Rep. 2017 Dec 8;7:17197. doi: 10.1038/s41598-017-17606-5 (PMC5722933; doi:10.1038/s41598-017-17606-5)
Supplement: Supplementary file 1 — Supplementary information [file 41598_2017_17606_MOESM1_ESM.pdf]

Supplementary information for:

Binding Modes of Teixobactin to Lipid II: Molecular Dynamics Study

Yang Liu[1], Yaxin Liu[1], Mary B. Chan-Park[2,3], and Yuguang Mu*[1]

[1]School of Biological Sciences, Nanyang Technological University (NTU), 60 Nanyang Drive, Singapore 637551

[2]School of Chemical and Biomedical Engineering, Nanyang Technological University (NTU), 62 Nanyang Drive, Singapore 637459

[3]Centre for Antimicrobial Bioengineering, NTU

Correspondence and requests for data should be addressed to Y.M. (email: ygmu@ntu.edu.sg)

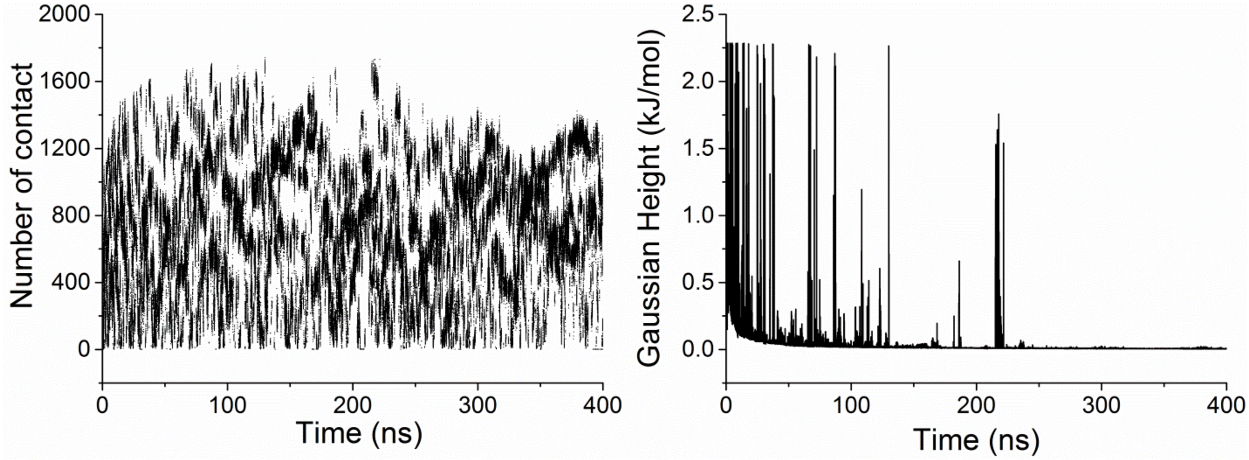

Figure S1 Diffusion of the number of contacts (left panel) and decay of Gaussian height (right panel) as a function of time during the PTMetaD-WTE simulation of TXB-L_{II} complex.

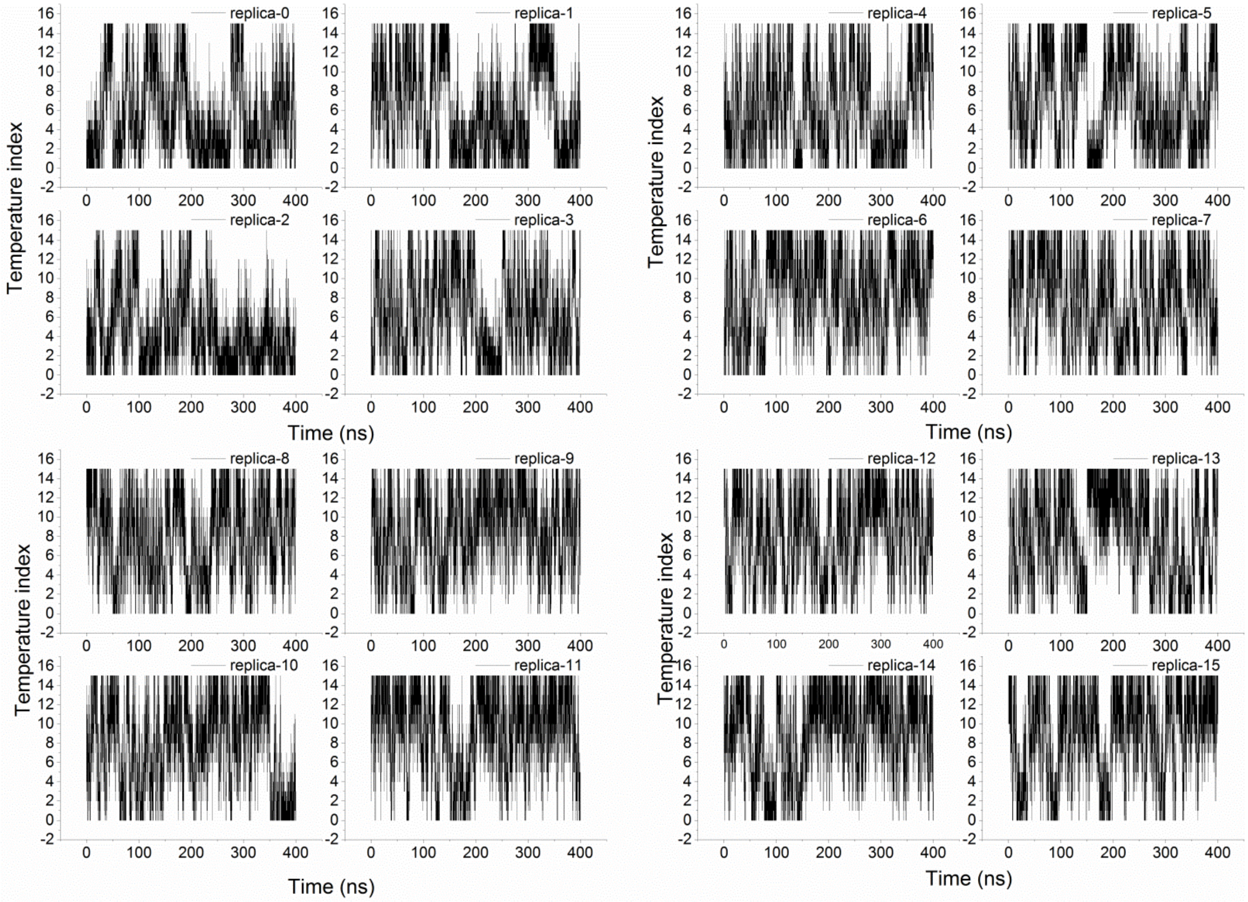

Figure S2 Diffusive movement of replicas in temperature space. Figure shows that our PTMetaD-WTE simulations for TXB-L_{II} complex are converged.

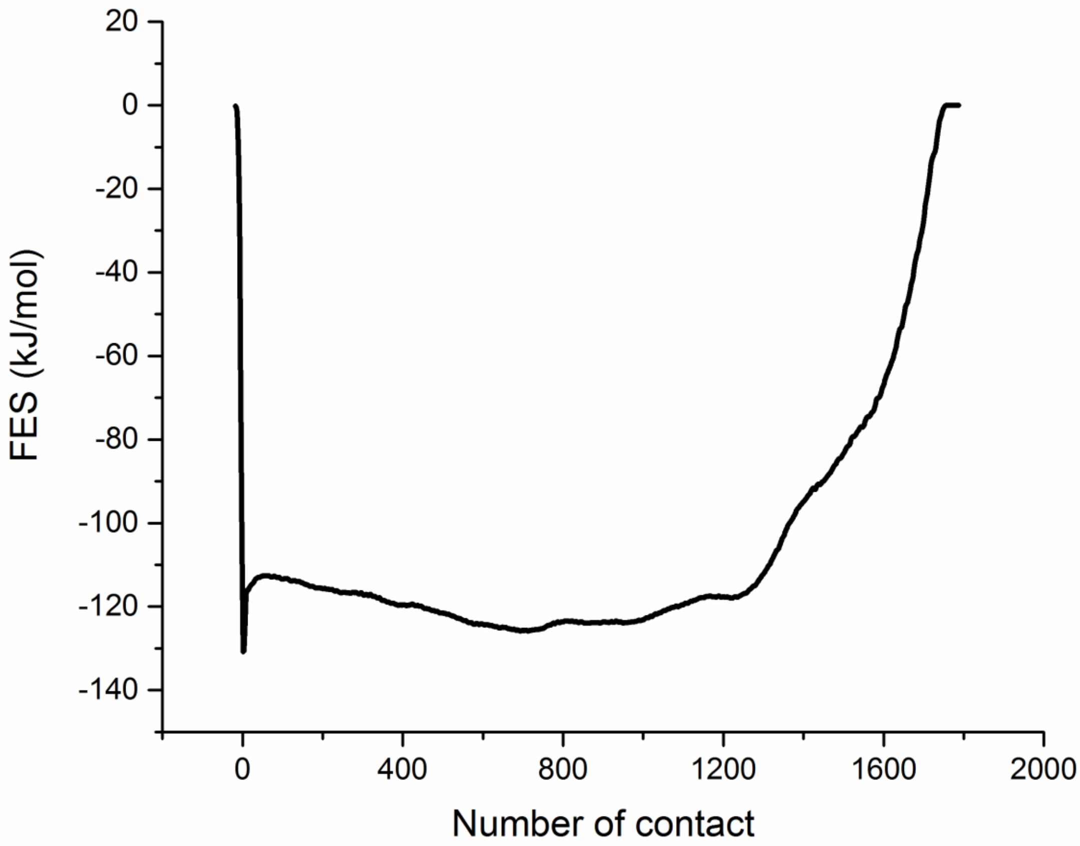

Figure S3 Free energy surface (FES) as a function of number of contacts. Curve was obtained by plumed tool sum_hills. No obvious local minima are found in FES.

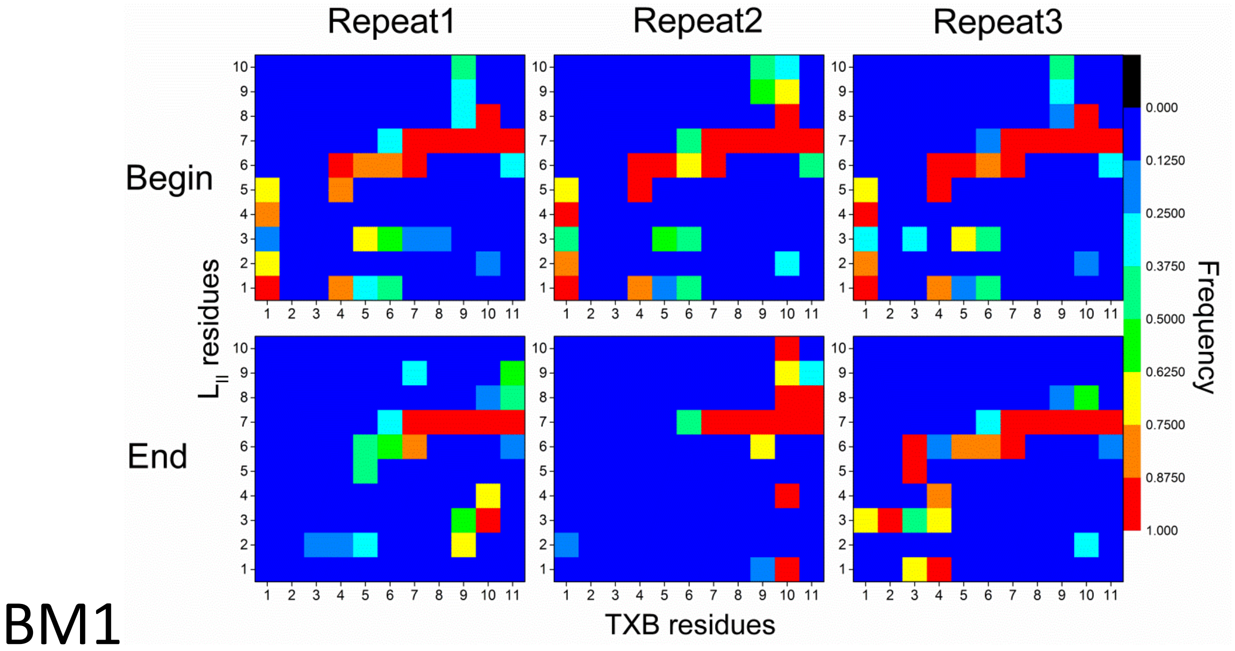

Repeat1
Repeat2
Repeat3
Begin
End
L_{II} residues
TXB residues
Frequency
0.000
0.1250
0.2500
0.3750
0.5000
0.6250
0.7500
0.8750
1.000
BM1

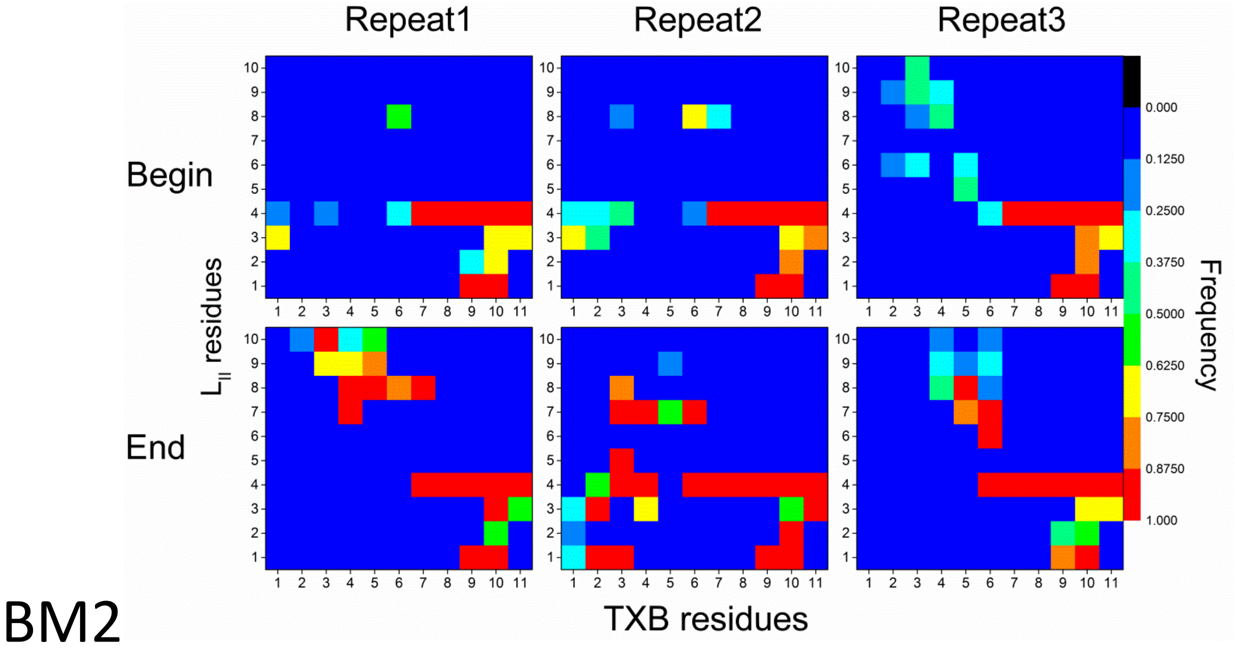

Repeat1
Repeat2
Repeat3
Begin
End
L_{II} residues
TXB residues
Frequency
0.000
0.1250
0.2500
0.3750
0.5000
0.6250
0.7500
0.8750
1.000
BM2

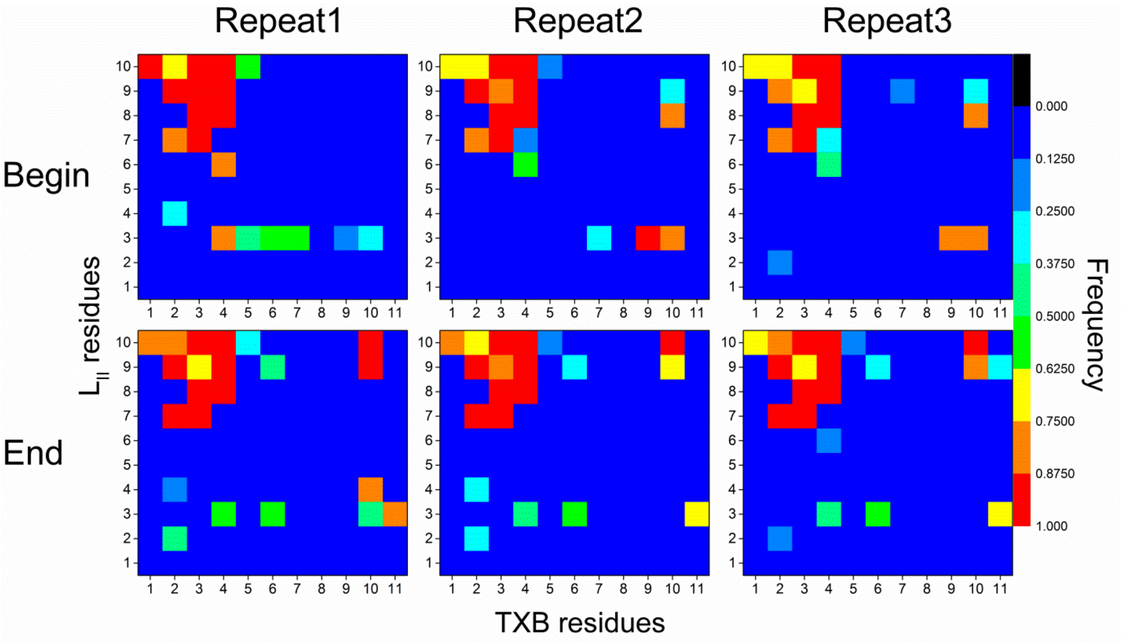

BM3

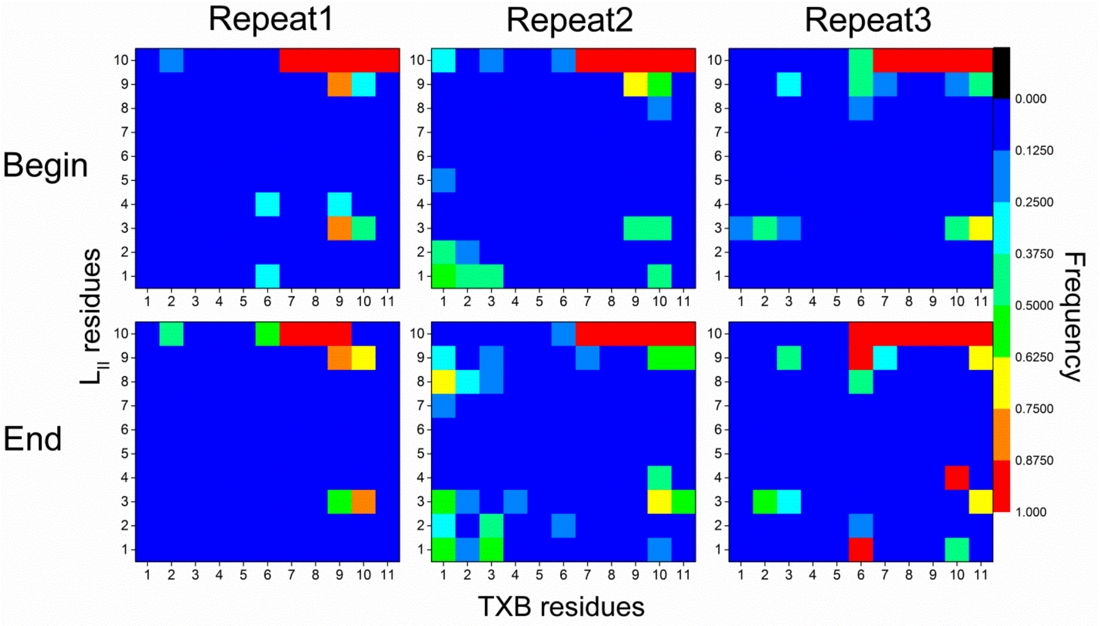

BM4

Figure S4 Residue based contact maps of first 10 ns simulation and last 10 ns for the four binding modes. Representative structures of each binding mode were simulated for 3 repeats with 200 ns each to check their stability. The contact cutoff was set as 0.4 nm and only heavy atoms were considered. The region of featured contacts for all the four binding modes have no changes when comparing the contact maps of the first 10 ns simulation and the last 10 ns simulation, indicating the strong stability of the four binding modes.

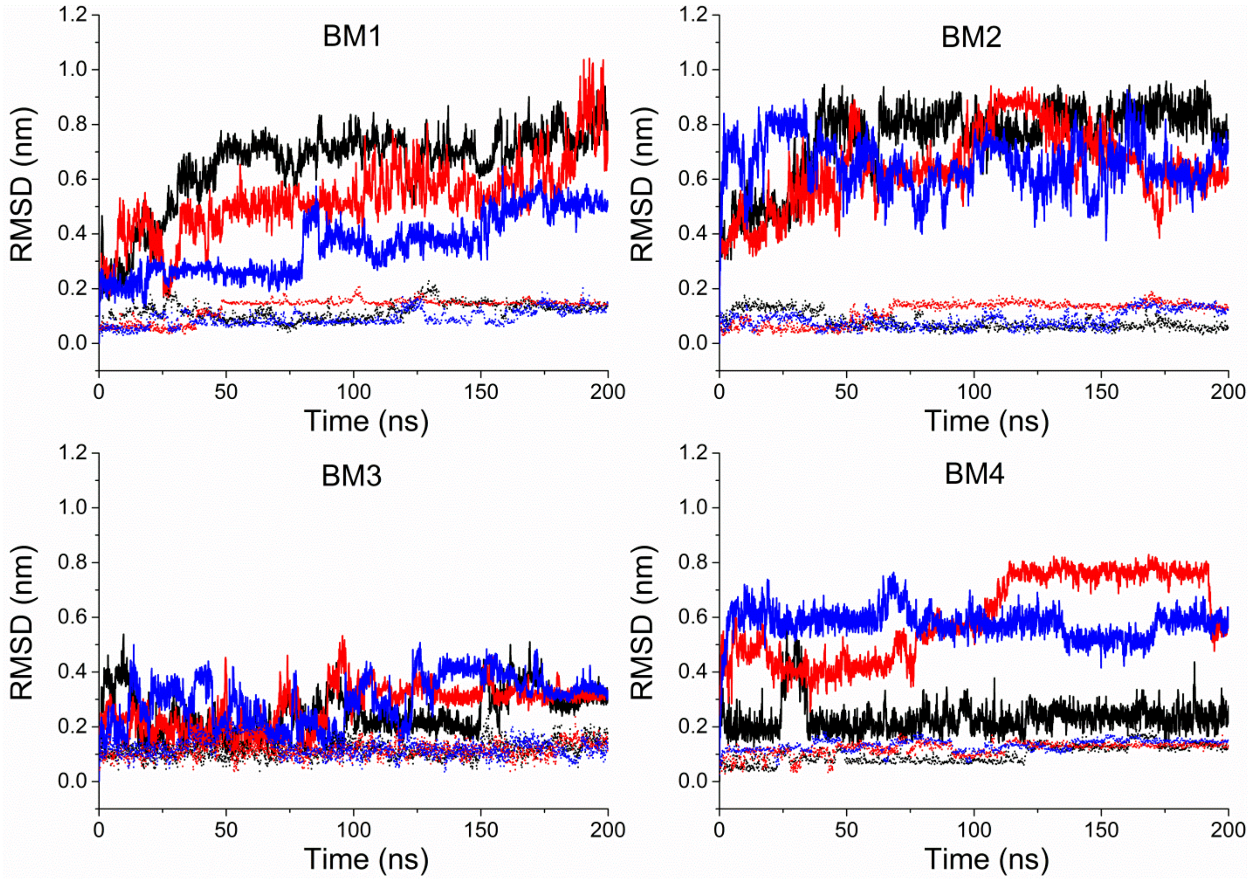

Figure S5 RMSD in the three repeats of stability-checking simulations for the four different binding modes. Black, red and blue colors represent repreat1, repeat2 and repeat3, respectively, and solid lines and dot lines represent the RMSD of whole complex (TXB and L_{II}) and RMSD of featured-contacting residues, respectively.

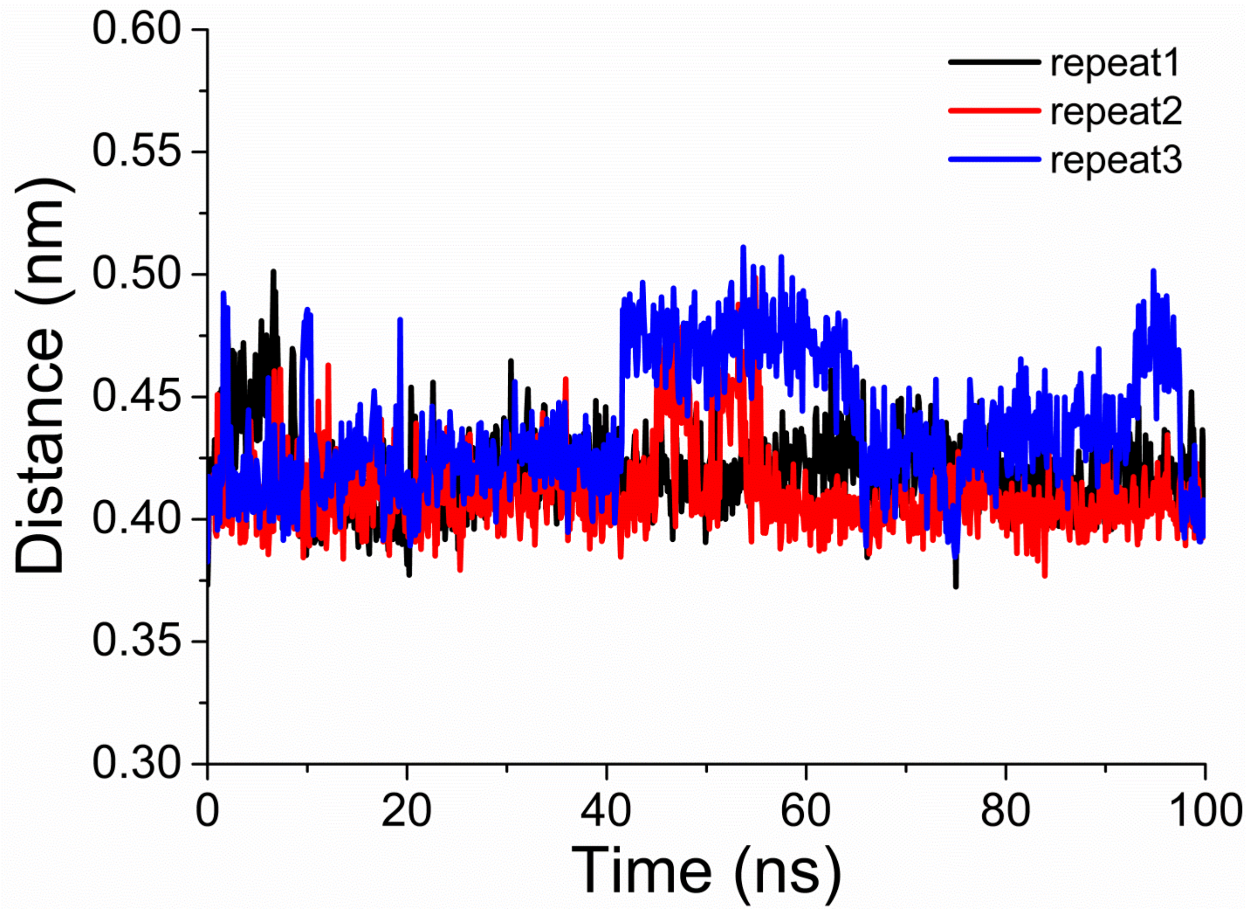

Figure S6 Three repeats of unbiased MD simulations (100 ns each) for nisin-L_{II} complex were performed to test the reliability of our force field parameters. Above figure shows the distance between the featured-contating residues, i.e. the ring motif of nisin and the pyrophosphate of L_{II}. According to our simulation, the binding for nisin and L_{II} shows high stability, indicating our force field parameters are reliable for the binding study.

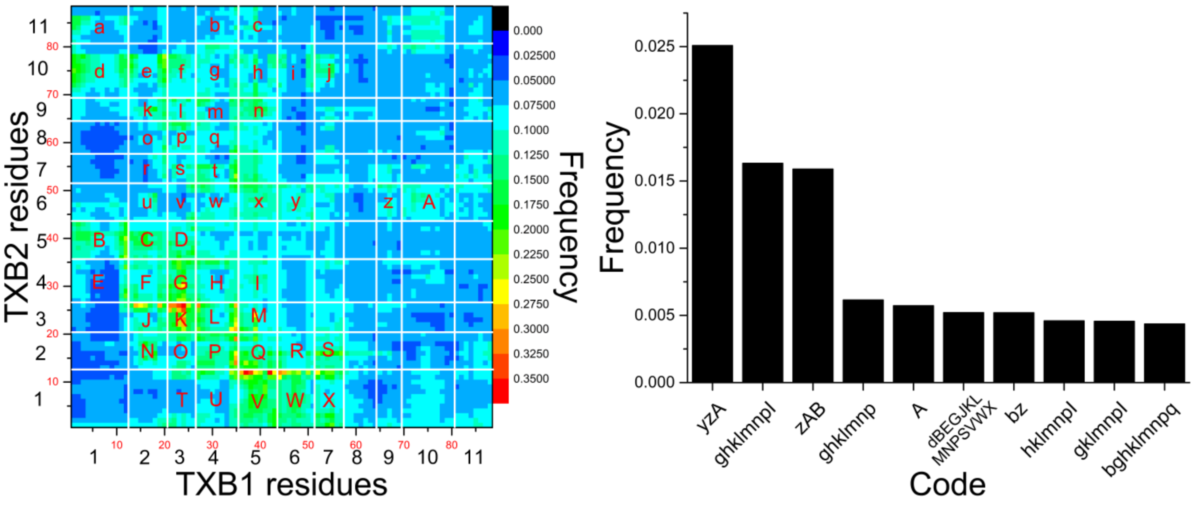

Fig S7 Study of TXB dimerization based on 400 ns PTMetaD-WTE simulation of two TXB molecules. This system had almost the same size as TXB- L_{II} system (2 TXB + 6510 H_2O + 21 Na^+ + 21 Cl^- versus 1 TXB + 1 L_{II} + 6506 H_2O + 24 Na^+ + 21 Cl^-), therefore we used the same parameters as those in the study of TXB- L_{II} binding. An atom-based contact map (left) was built and atom-pairs with high contacting frequencies were labelled with different single letters. We then grouped the labelled frames, and made a histogram of different groups (right panel showed ten groups with the highest frequencies). We chose the first two groups (group yzA and group ghklmnpl) to do the further analysis, since they have common features with the following groups. Group yzA has featured-contacts between the ring motif of TXB1 and Ile-6 of TXB2, and group ghklmnpl has featured-contacts between Ser-3-Gln-4 segment of TXB1 and ring motif of TXB2.

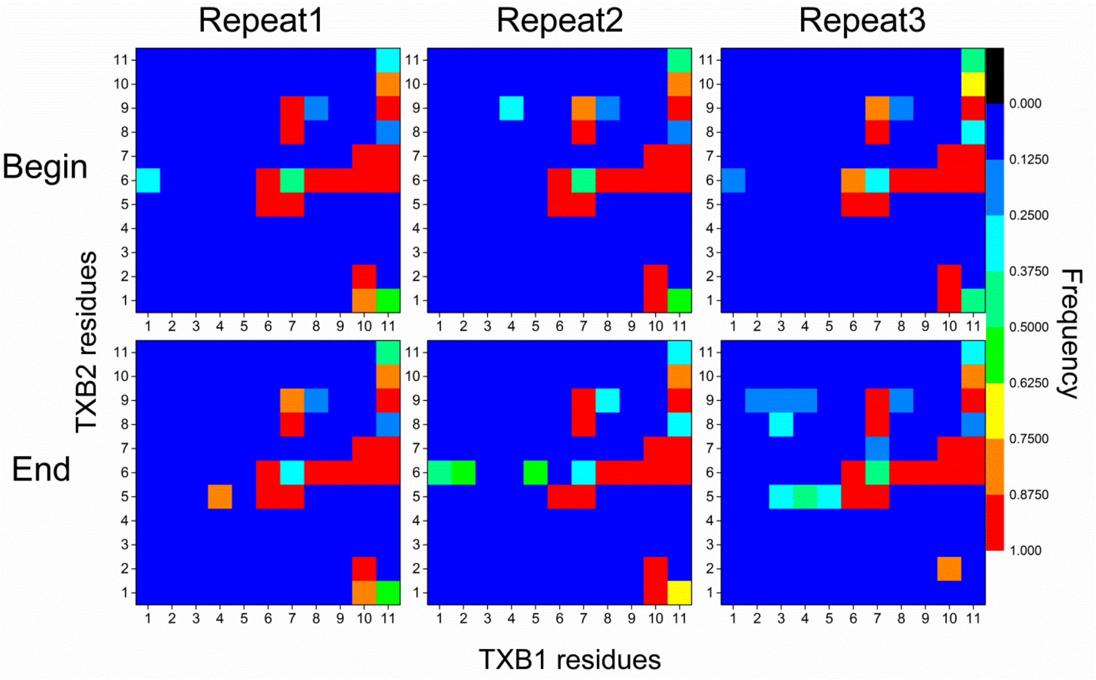

yzA

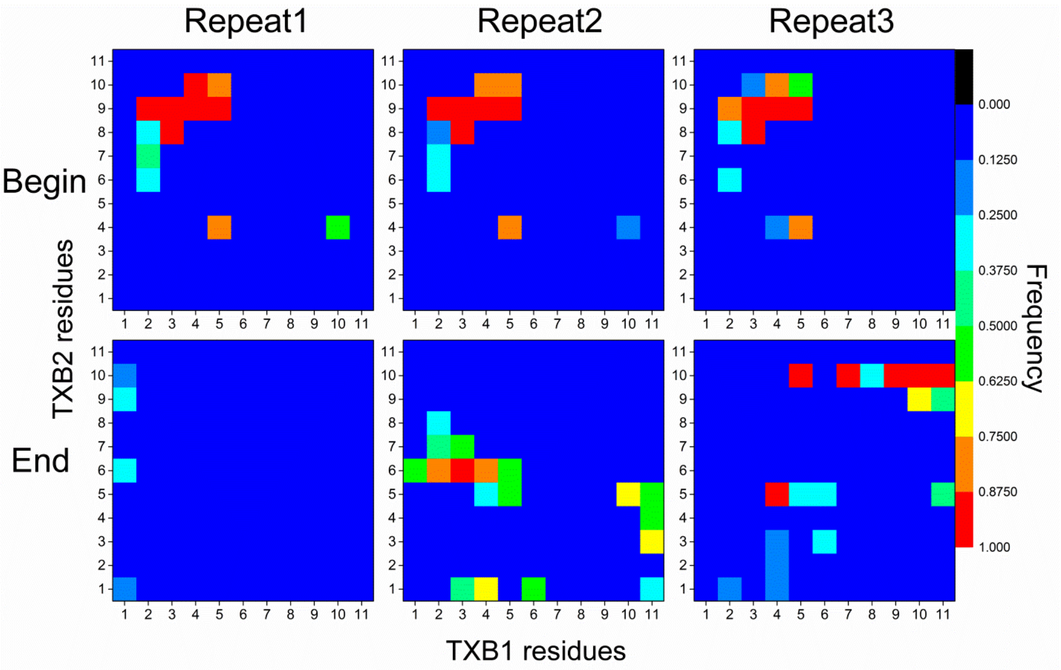

ghklmnpl

Figure S8 Starting from the representive structures of group yzA and group ghklmnpl, three repeats of unbiased MD simulations, 200 ns each, were performed to check the stabilities of these two groups. Contact maps between two TXB molecules of the first 10 ns and last 10 ns trajectory for each simulation were compared, and only featured-contacts of group yzA could be maintained during the simulation. Group ghklmnpl is not stable.

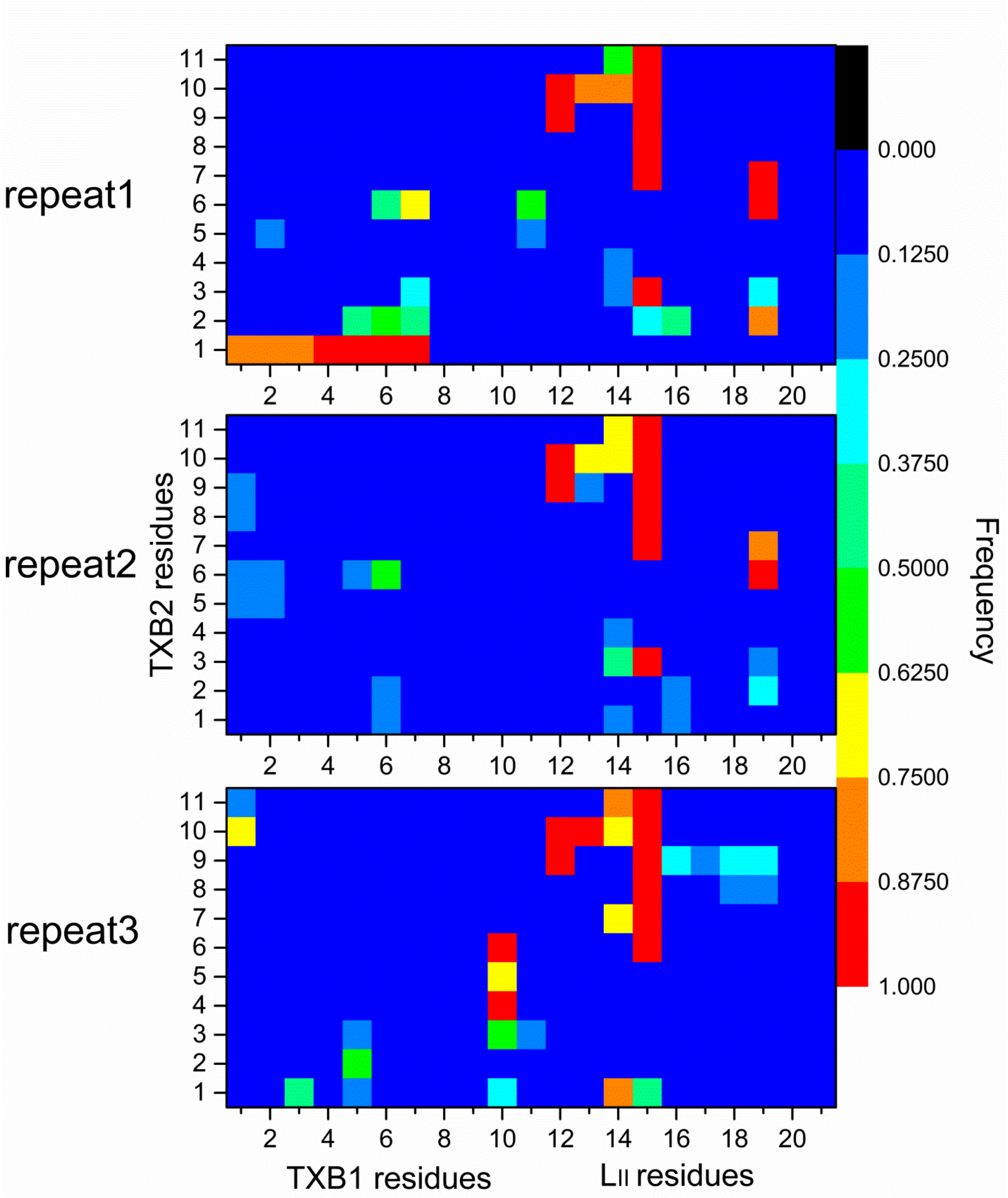

Figure S9 A L_{II} molecule was then inserted into the conformation of group yzA to form a 2:1 TXB-L_{II} complex. This new complex, which had the pyrophosphate group of L_{II} H bonding with the free ring-motif of TXB2, was used as an initial structure to produce another series of unbiased MD simulation (three repeats with 200 ns each). Above figures presents the contacts inside the complex based on last 50 ns trajectories, and the featured contacts between two TXB moleculs are disappeared. These results reveal that TXB molecules form stable dimer in solution, however, the dimer could not be maintained with the competition of L_{II} molecule.

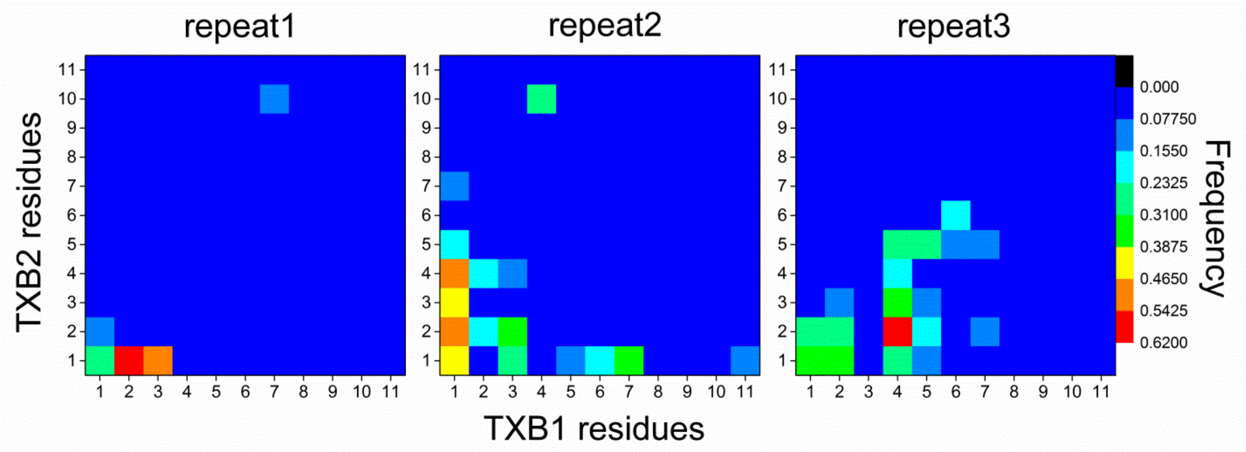

Figure S10 Contact map between two TXB molecules in the TXB-L_{II} complex with a ratio of 2:1 (BM1&BM2 system). Last 50 ns simulation was used to generate the contact map. No specific binding pattern is found over the three repeats.

Table S1 Codes for frames with different contacting patterns and their frequencies (TXB-L_{II})

Code	Frequency	Code	Frequency	Code	Frequency	Code	Frequency
opqrst	9.81E-02	t	2.91E-03	ruvwx	1.03E-03	klmnqruvwx	5.03E-04
uvwxy	7.39E-02	bcdefgh	2.81E-03	nopqrs	9.61E-04	h	5.01E-04
abcdefgh	6.73E-02	agopqrst	2.80E-03	rx	9.35E-04	eg	4.99E-04
jklmn	6.17E-02	copqrsy	2.78E-03	q	8.82E-04	bo	4.85E-04
iuvwxy	4.82E-02	brsx	2.71E-03	gjklmn	8.75E-04	grx	4.75E-04
opqrs	3.54E-02	fjklmn	2.69E-03	lopqrst	8.71E-04	ipquvwxy	4.71E-04
abcefgh	1.89E-02	bcefgh	2.63E-03	iuvwx	8.56E-04	auvwxy	4.67E-04
jklmnx	1.59E-02	ops	2.45E-03	cuvwxy	8.22E-04	mopqrs	4.65E-04
iopqrst	1.37E-02	ajkl	2.39E-03	k	8.11E-04	ho	4.64E-04
fijklmn	1.29E-02	efgopqrs	2.34E-03	nopqrstxy	7.89E-04	bcefghj	4.64E-04
abdgopqrst	1.28E-02	o	2.33E-03	eop	7.60E-04	abcdfgh	4.64E-04
jkl	1.25E-02	fgopqrs	2.26E-03	beg	7.59E-04	ikluvwxy	4.62E-04
opqrstx	1.16E-02	opqrsty	2.20E-03	hx	7.50E-04	abefghuwx	4.55E-04
jklmnw	1.04E-02	ilmuvwxy	2.17E-03	dgopqrst	7.43E-04	rxy	4.55E-04
adgopqrst	9.36E-03	mopqrstu	2.10E-03	ilmquvwx	7.37E-04	fghopqrs	4.55E-04
j	7.33E-03	opqrsy	2.08E-03	ab	7.36E-04	op	4.53E-04
abdegopqrst	7.27E-03	uv	1.95E-03	bemnx	7.17E-04	bcdefghj	4.53E-04
jklmny	7.10E-03	abefgh	1.89E-03	ilmuvwx	7.03E-04	iklmuvwxy	4.52E-04
acduvwy	7.04E-03	iopqrs	1.87E-03	fijkl	6.77E-04	copqrstx	4.48E-04
u	7.04E-03	egiuvwxy	1.86E-03	ai	6.67E-04	du	4.45E-04
y	6.86E-03	abrsx	1.84E-03	wxy	6.62E-04	hopqrst	4.34E-04
uvwx	6.45E-03	begmx	1.67E-03	abegopqrst	6.59E-04	grsx	4.32E-04
cdfghuvwxy	5.85E-03	huvwxy	1.64E-03	jopqrs	6.56E-04	adi	4.26E-04
xy	5.58E-03	beop	1.62E-03	my	6.48E-04	gopqrsy	4.26E-04
opqrsx	5.43E-03	qruvwx	1.60E-03	uvw	6.41E-04	bop	4.24E-04
opqrstu	5.39E-03	jklw	1.55E-03	gu	6.37E-04	jklmnu	4.20E-04
bopqrst	5.33E-03	beo	1.52E-03	abcdefghuwx	6.25E-04	p	4.16E-04
gopqrst	5.21E-03	ac	1.45E-03	fiopqrst	6.24E-04	abcefghj	4.11E-04
rsx	5.06E-03	aopqrsty	1.39E-03	mruvwx	6.22E-04	bem	4.07E-04
x	4.72E-03	iquvwxy	1.38E-03	lmopqrstu	6.13E-04	bjx	4.05E-04
gopqrs	4.62E-03	g	1.35E-03	jkln	6.10E-04	mn	4.00E-04
a	4.51E-03	ikuvwxy	1.30E-03	nuvwxy	6.08E-04	befgh	3.98E-04
opqrstxy	4.37E-03	gopqrsxy	1.30E-03	iy	6.01E-04	bopqrsty	3.92E-04
adegopqrst	4.22E-03	nopqrsy	1.29E-03	w	5.86E-04	jklm	3.87E-04
abcdefghj	4.14E-03	gopqrstx	1.26E-03	n	5.70E-04	fghiuvwxy	3.86E-04
mopqrst	3.81E-03	abgopqrst	1.26E-03	ipuvwxy	5.66E-04	bemn	3.82E-04
hjklmn	3.75E-03	imuvwxy	1.23E-03	iluvwx	5.54E-04	tuvwxy	3.81E-04
c	3.74E-03	opqrsxy	1.21E-03	cq	5.50E-04	jl	3.73E-04
ijklmn	3.71E-03	ix	1.20E-03	bey	5.44E-04	klmqruvwx	3.72E-04
egopqrst	3.52E-03	v	1.19E-03	fghiquvwxy	5.43E-04	copqrstu	3.67E-04
i	3.36E-03	bei	1.19E-03	copqrst	5.43E-04	opqrsu	3.65E-04
bemx	3.29E-03	abdefgh	1.19E-03	abcfgh	5.42E-04	ij	3.59E-04
lopqrs	3.21E-03	acduvw	1.18E-03	adgopqrs	5.41E-04	eo	3.58E-04
iluvwxy	3.13E-03	r	1.18E-03	bx	5.35E-04	bcemnx	3.55E-04
b	3.12E-03	arsx	1.09E-03	cmx	5.34E-04	cdfhuvwxy	3.52E-04
be	3.05E-03	ejklmn	1.07E-03	iu	5.29E-04	nopqrst	3.46E-04
ixy	3.01E-03	egopqrs	1.05E-03	aegopqrst	5.25E-04	begmnx	3.45E-04
iopquvwxy	2.98E-03	abcdegh	1.05E-03	qrst	5.22E-04	egh	3.44E-04
muvwxy	2.96E-03	dfh	1.03E-03	abcefg	5.16E-04	adfguvwxy	3.38E-04
copqrsxy	2.93E-03	m	1.03E-03	mx	5.14E-04	begi	3.38E-04

Code	Frequency	Code	Frequency	Code	Frequency	Code	Frequency
ad	3.32E-04	bcemx	2.11E-04	egiopqrst	1.46E-04	sxy	1.11E-04
cdefgh	3.16E-04	jopqrsxy	2.06E-04	cjl	1.45E-04	abefghuw	1.09E-04
cdfuvwx	3.16E-04	egopqrstx	2.04E-04	ijklm	1.44E-04	cdu	1.09E-04
arx	3.15E-04	aefguvwxy	1.99E-04	cgopqrst	1.44E-04	gxy	1.08E-04
pu	3.13E-04	guvwxy	1.99E-04	wy	1.42E-04	cefgh	1.07E-04
ben	3.10E-04	fguvwxy	1.97E-04	bery	1.41E-04	lmuvwxy	1.07E-04
oqrst	3.09E-04	emn	1.97E-04	hoq	1.41E-04	fg	1.07E-04
e	3.07E-04	afguvwxy	1.95E-04	egopqrsx	1.40E-04	orst	1.05E-04
adefguvwxy	3.06E-04	klmruvwx	1.94E-04	begy	1.40E-04	abceghj	1.03E-04
abcefghuwx	3.06E-04	vx	1.94E-04	iklmpuvwxy	1.39E-04	abcefghi	1.03E-04
gopqrsx	3.05E-04	eh	1.94E-04	bmx	1.37E-04	kv	1.03E-04
fgopqrsxy	3.02E-04	gy	1.93E-04	pqu	1.37E-04	cgopqrstu	1.01E-04
imquvwx	2.94E-04	beiopqrst	1.92E-04	acdefguvwxy	1.35E-04	mopqrstxy	1.01E-04
cjklmn	2.91E-04	befghuwx	1.92E-04	begn	1.35E-04	bjklmnt	9.98E-05
ost	2.90E-04	ceghopq	1.91E-04	mnuvwxy	1.35E-04	cg	9.91E-05
gopqrstu	2.89E-04	acdefgh	1.89E-04	imuvwx	1.33E-04	hiopqrst	9.87E-05
chouvwxy	2.87E-04	biopqrst	1.87E-04	abcegh	1.30E-04	iouvwxy	9.86E-05
fgopqrsx	2.85E-04	eopqrs	1.86E-04	brx	1.30E-04	eopqrstx	9.79E-05
qu	2.83E-04	uwxy	1.84E-04	bcegh	1.29E-04	fijklm	9.76E-05
abcdefghw	2.81E-04	lxy	1.84E-04	py	1.27E-04	df	9.67E-05
bgopqrst	2.80E-04	kmqruvwx	1.84E-04	abcefghw	1.27E-04	abx	9.60E-05
kmnqruvwx	2.74E-04	ajklw	1.81E-04	copqrsu	1.26E-04	begmn	9.48E-05
by	2.73E-04	qrx	1.81E-04	bopqrstxy	1.26E-04	bceg	9.45E-05
cgopqrstx	2.72E-04	dfu	1.79E-04	defguvwxy	1.25E-04	abgopqrs	9.40E-05
cfhouvwxy	2.72E-04	ilmquvwxy	1.78E-04	abdgopqrs	1.25E-04	beopqrst	9.36E-05
fh	2.69E-04	fijkln	1.77E-04	cuvwx	1.25E-04	ilmpuvwxy	9.31E-05
buvwxy	2.67E-04	ber	1.77E-04	degopqrst	1.24E-04	agopqrs	9.30E-05
adegopqrs	2.67E-04	fho	1.77E-04	iopqrsty	1.23E-04	jklnw	9.22E-05
bg	2.64E-04	fiopqrs	1.76E-04	klmnruvwx	1.23E-04	giopqrst	9.01E-05
lopqrstu	2.64E-04	abcdefg	1.73E-04	klmn	1.23E-04	begr	8.96E-05
mnopqrstu	2.60E-04	bxy	1.69E-04	impquvwx	1.22E-04	ijklmnw	8.96E-05
fgopqrsy	2.60E-04	bgrsx	1.69E-04	fm	1.21E-04	bopqrstx	8.93E-05
ghj	2.59E-04	cj	1.69E-04	defh	1.21E-04	degh	8.86E-05
adu	2.59E-04	fuvwxy	1.66E-04	gx	1.20E-04	adfgopqrst	8.84E-05
hopqrstxy	2.57E-04	mqruvwx	1.64E-04	opqrt	1.19E-04	bix	8.75E-05
bce	2.57E-04	lsxy	1.64E-04	cfuvwx	1.18E-04	abfgh	8.74E-05
iklmnuvwxy	2.52E-04	bi	1.64E-04	ijkl	1.18E-04	cm	8.69E-05
ijuvwxy	2.46E-04	gh	1.62E-04	ot	1.18E-04	aijkl	8.66E-05
jlopqrs	2.45E-04	gho	1.59E-04	gijklmn	1.18E-04	aci	8.65E-05
bijx	2.43E-04	gors	1.58E-04	cu	1.17E-04	egopqrstu	8.63E-05
gmopqrstxy	2.41E-04	kmnruvwx	1.57E-04	beguvwxy	1.16E-04	begry	8.61E-05
bopqrs	2.35E-04	ajkln	1.57E-04	copqrs	1.16E-04	acdfgh	8.54E-05
abdegopqrs	2.33E-04	ax	1.55E-04	chjklmn	1.16E-04	adfg	8.47E-05
st	2.33E-04	pqrst	1.54E-04	acdgopqrst	1.16E-04	qrs	8.46E-05
nopqrsty	2.30E-04	abcdefghu	1.53E-04	uvwy	1.15E-04	lmopqrs	8.40E-05
iopuvwxy	2.20E-04	f	1.52E-04	lmx	1.14E-04	abdefghuwx	8.32E-05
abcdefghi	2.14E-04	jklmnt	1.51E-04	fhiquvwxy	1.14E-04	bgmx	8.30E-05
fopqrs	2.14E-04	gix	1.50E-04	jklxy	1.14E-04	ajklmnw	8.16E-05
s	2.12E-04	iklmnpuvwxy	1.48E-04	ijkuvwxy	1.13E-04	iklmpquvwxy	8.15E-05
eopqrst	2.11E-04	abd	1.47E-04	wx	1.13E-04	fjkl	8.14E-05

Code	Frequency	Code	Frequency	Code	Frequency	Code	Frequency
gopqrstxy	8.00E-05	bceop	6.17E-05	bm	4.80E-05	adefg	3.70E-05
bfgh	7.97E-05	adeu	6.10E-05	lx	4.78E-05	fi	3.69E-05
adegopqrt	7.89E-05	opsx	6.07E-05	acg	4.77E-05	cduvwx	3.67E-05
abrx	7.85E-05	acdjuvwy	6.06E-05	bdgopqrst	4.75E-05	bep	3.66E-05
kvx	7.85E-05	abcdefghuw	6.04E-05	gnopqrs	4.75E-05	kot	3.66E-05
bcfgh	7.78E-05	buvwy	6.02E-05	abcdefghjw	4.73E-05	bcmx	3.66E-05
abceghi	7.78E-05	luvwxy	5.99E-05	gor	4.67E-05	fopqrstu	3.65E-05
opqrtx	7.77E-05	deguvwxy	5.92E-05	ort	4.62E-05	pqrs	3.64E-05
inopqrst	7.76E-05	jkxy	5.85E-05	dfpu	4.62E-05	hopqrs	3.61E-05
rst	7.72E-05	jqrs	5.71E-05	eghjopqs	4.59E-05	ilopquvwxy	3.60E-05
eghopq	7.72E-05	dfguvwxy	5.67E-05	jlqrs	4.58E-05	bdefgh	3.59E-05
prs	7.66E-05	deg	5.66E-05	nrxy	4.58E-05	absx	3.59E-05
abeghuwx	7.66E-05	eklmqwxy	5.61E-05	ilquvwx	4.56E-05	adfu	3.54E-05
nopqrstu	7.59E-05	klm	5.56E-05	bcd	4.56E-05	ju	3.51E-05
bij	7.53E-05	jmr	5.56E-05	fjklm	4.52E-05	ruvwxy	3.50E-05
au	7.53E-05	jnopqrsy	5.55E-05	bijklmnt	4.48E-05	dfhuvwxy	3.49E-05
abdi	7.38E-05	bnx	5.50E-05	ny	4.46E-05	abcdfghj	3.46E-05
lopqrsy	7.28E-05	imopqrst	5.48E-05	bceghi	4.43E-05	dfhopqrstu	3.46E-05
ty	7.25E-05	cjklmnw	5.45E-05	bopqrstw	4.41E-05	dh	3.44E-05
fopqrsy	7.23E-05	ijkln	5.45E-05	abcfghj	4.41E-05	bgy	3.42E-05
gmo	7.22E-05	beijx	5.39E-05	eghj	4.38E-05	defhopqs	3.42E-05
ghiopqrst	7.20E-05	buvw	5.37E-05	klopqrs	4.34E-05	jnopqrs	3.40E-05
hxy	7.19E-05	bgi	5.36E-05	eghiopqrst	4.33E-05	jps	3.35E-05
mo	7.15E-05	kn	5.34E-05	gmnopqrstxy	4.27E-05	cghopq	3.34E-05
go	7.12E-05	bj	5.34E-05	defg	4.26E-05	ps	3.32E-05
eu	7.04E-05	bejx	5.34E-05	nuv	4.25E-05	ghopqrs	3.32E-05
sx	6.92E-05	aefguvwx	5.31E-05	aopqrst	4.24E-05	ctuvwxy	3.32E-05
beu	6.87E-05	gry	5.27E-05	km	4.23E-05	dfghuvwxy	3.28E-05
bcdefghw	6.85E-05	defghuvwxy	5.27E-05	jpqrs	4.22E-05	afg	3.26E-05
fhiuvwxy	6.84E-05	d	5.27E-05	abefghux	4.17E-05	egr	3.26E-05
euvwxy	6.82E-05	beopqrs	5.24E-05	ex	4.15E-05	aix	3.24E-05
acuvwy	6.80E-05	abcdeghi	5.22E-05	begopqrst	4.08E-05	ijklmnt	3.23E-05
abeopqrst	6.79E-05	jlpqrs	5.21E-05	efghopq	4.08E-05	gjklmno	3.22E-05
cdghuvwxy	6.77E-05	abdefghux	5.21E-05	egmopqt	4.07E-05	efgopq	3.21E-05
cdfgh	6.73E-05	gjopqrs	5.17E-05	begiopqrst	4.07E-05	fijklmnu	3.17E-05
fqu	6.72E-05	eghjopq	5.15E-05	bmn	4.07E-05	bjklmn	3.10E-05
hopq	6.71E-05	iquvwx	5.13E-05	fopqrsxy	4.05E-05	bcmnx	3.10E-05
bgopqrstx	6.65E-05	bemy	5.12E-05	bnopqrstxy	4.02E-05	fhoq	3.10E-05
jklmnxy	6.63E-05	pr	5.11E-05	hn	3.94E-05	abdfgopqrst	3.08E-05
fopqrsx	6.63E-05	mnopqrs	5.10E-05	bgopqrs	3.91E-05	hmn	3.07E-05
ah	6.60E-05	befghj	5.09E-05	abdegopqrt	3.88E-05	acx	3.03E-05
lqrst	6.57E-05	begopqrs	5.06E-05	lmqruvwx	3.84E-05	eguvwxy	3.02E-05
hopqrsty	6.54E-05	ghopq	5.05E-05	bwx	3.81E-05	fhopqrstu	3.02E-05
iotuvwx	6.54E-05	kqruvwx	5.04E-05	otuvwx	3.80E-05	abcd	3.02E-05
aopqrstx	6.46E-05	abegopqrstx	5.03E-05	bceh	3.75E-05	gmopqrsxy	3.00E-05
efgopqrstx	6.42E-05	most	4.99E-05	bn	3.74E-05	egopq	2.99E-05
dfgh	6.40E-05	bmnx	4.97E-05	acdegopqrs	3.73E-05	fv	2.98E-05
qr	6.38E-05	jk	4.93E-05	tuvwx	3.72E-05	abdgopqrstu	2.96E-05
duvwxy	6.38E-05	huvw	4.86E-05	bgn	3.71E-05	ghrsx	2.93E-05
knopqrs	6.22E-05	chuvwxy	4.83E-05	hm	3.70E-05	beny	2.93E-05

Code	Frequency	Code	Frequency	Code	Frequency	Code	Frequency
abdeopqrt	2.92E-05	efguvwxy	2.07E-05	acdjuvw	1.52E-05	iopqrt	1.10E-05
hq	2.86E-05	jo	2.07E-05	eghuvwxy	1.52E-05	beopqrstu	1.10E-05
mst	2.83E-05	dhopqrstu	2.07E-05	defgopqrs	1.52E-05	ioptuvwx	1.09E-05
cjkl	2.81E-05	amx	2.06E-05	bgopqrstxy	1.51E-05	beuwx	1.09E-05
flm	2.81E-05	hiopqrsty	2.06E-05	cd	1.51E-05	ilmpquvwxy	1.09E-05
hmopqrst	2.77E-05	ilmpquvwx	2.04E-05	ipquvwx	1.51E-05	iklmnpquvwxy	1.08E-05
fvx	2.76E-05	em	2.04E-05	fgnopqrstu	1.50E-05	chjopq	1.08E-05
bc	2.75E-05	ijx	2.04E-05	giopqrs	1.48E-05	axy	1.07E-05
cfgh	2.73E-05	cfghjopqs	2.04E-05	efghuvwxy	1.46E-05	klmnw	1.07E-05
ag	2.72E-05	ghopqs	2.03E-05	impquvwxy	1.46E-05	bewx	1.07E-05
beixy	2.70E-05	bcey	2.03E-05	imnopqrst	1.46E-05	cdfhuvwx	1.06E-05
bceo	2.69E-05	ilpquvwxy	2.00E-05	klxy	1.43E-05	hko	1.06E-05
dm	2.68E-05	ilquvwxy	2.00E-05	jp	1.43E-05	fijklmny	1.06E-05
quvwx	2.63E-05	mrt	1.99E-05	abeopqrstu	1.42E-05	eiopqrst	1.06E-05
bopqrx	2.63E-05	defhuvwxy	1.98E-05	fgho	1.42E-05	adefu	1.05E-05
deuvwxy	2.62E-05	houvwxy	1.97E-05	fhuvwxy	1.40E-05	beiopqrsty	1.04E-05
kt	2.62E-05	abe	1.95E-05	defuvwxy	1.39E-05	abdeopqrstu	1.03E-05
jops	2.60E-05	bexy	1.92E-05	ej	1.39E-05	abefguvwx	1.03E-05
nxy	2.60E-05	iklnuvwxy	1.91E-05	ajklnw	1.39E-05	lmopqrst	1.03E-05
iotuvwxy	2.54E-05	cdx	1.83E-05	abcdefghux	1.38E-05	fmn	9.99E-06
bfopqrstu	2.53E-05	lpqrst	1.83E-05	beuvwxy	1.36E-05	iopquvwx	9.73E-06
mt	2.51E-05	ciotuvwx	1.82E-05	gr	1.32E-05	knopqrsy	9.64E-06
adefguvwx	2.50E-05	gopqrt	1.80E-05	opqt	1.29E-05	ouvwx	9.37E-06
rsxy	2.49E-05	opqrstwy	1.76E-05	bgopqrstw	1.28E-05	fghopqrstu	9.33E-06
bcdegh	2.48E-05	adeguvwxy	1.74E-05	asxy	1.28E-05	ilouvwxy	9.32E-06
bgiopqrst	2.46E-05	abdfopqrstu	1.74E-05	vw	1.27E-05	cghjopq	9.32E-06
acdi	2.45E-05	abdegh	1.74E-05	iopuvwx	1.27E-05	afopqrstu	9.26E-06
bex	2.42E-05	abegh	1.73E-05	lmsxy	1.26E-05	bopqrsy	9.23E-06
dfg	2.41E-05	ghjopq	1.73E-05	abopqrstxy	1.26E-05	acdx	9.17E-06
bco	2.41E-05	ioprst	1.73E-05	kmn	1.26E-05	egij	9.15E-06
adegopq	2.38E-05	bijklmn	1.72E-05	abdu	1.25E-05	ry	9.10E-06
cfgopqrs	2.37E-05	adijkl	1.70E-05	adefgopqrst	1.20E-05	adopqrstu	9.09E-06
ciu	2.35E-05	fgmopqrstu	1.69E-05	fgiopqrs	1.18E-05	mnopqrsty	9.03E-06
clmx	2.34E-05	klmquvwx	1.68E-05	imquvwxy	1.17E-05	fgopqrstuv	8.94E-06
bcdx	2.32E-05	abfopqrstu	1.67E-05	fopqrstuv	1.17E-05	ilopqrst	8.94E-06
adfopqrstu	2.31E-05	iklmquvwxy	1.66E-05	cmn	1.16E-05	pv	8.89E-06
ouvwxy	2.31E-05	opst	1.66E-05	fghuvwxy	1.16E-05	egopqrstuv	8.77E-06
acduvwxy	2.29E-05	fgopqrstu	1.64E-05	cdfghuvwx	1.16E-05	ciotuvwxy	8.75E-06
bopqrt	2.29E-05	lv	1.61E-05	abcdefh	1.15E-05	abjx	8.71E-06
gnopqrstxy	2.28E-05	br	1.61E-05	beghiopqrst	1.15E-05	nry	8.71E-06
deghuvwxy	2.26E-05	egu	1.61E-05	gnopqrstu	1.15E-05	egijl	8.66E-06
jlqrst	2.24E-05	ceghjopqs	1.60E-05	fhouvwxy	1.14E-05	ey	8.66E-06
l	2.24E-05	begopqrstu	1.60E-05	rt	1.14E-05	cfghopq	8.54E-06
befg	2.24E-05	cituvwxy	1.60E-05	cotuvwxy	1.14E-05	abegopqrstu	8.46E-06
gmopqrstu	2.21E-05	hjo	1.57E-05	hj	1.14E-05	bdefopqrstu	8.37E-06
ipqrst	2.20E-05	defhopqrstu	1.56E-05	bcdefghu	1.13E-05	bgopqrstuv	8.33E-06
aguvwxy	2.19E-05	bpqrst	1.56E-05	nv	1.13E-05	hopqrstu	8.30E-06
uxy	2.18E-05	adopqrst	1.54E-05	opqr	1.13E-05	fhmopqrstu	8.27E-06
jx	2.12E-05	mopqrsty	1.53E-05	hjko	1.12E-05	ioquvwxy	8.13E-06
afuvwxy	2.11E-05	rs	1.53E-05	aoqrtu	1.10E-05	bcde	7.97E-06

Code	Frequency	Code	Frequency	Code	Frequency	Code	Frequency
mort	7.84E-06	bcdefghuwx	5.27E-06	bfgmopqrstu	3.09E-06	mopqrsu	2.02E-06
acefguvwxy	7.80E-06	abi	5.19E-06	eghopqrsty	3.00E-06	beghiopqrsy	2.01E-06
abgopqrstu	7.78E-06	imopqrs	5.10E-06	inopqrs	2.97E-06	jklmx	1.99E-06
cdefghopq	7.75E-06	fhopqrstuv	5.09E-06	bceguvwxy	2.96E-06	aoqrtuv	1.97E-06
fx	7.73E-06	acdegopqrst	5.07E-06	adegopqrstu	2.94E-06	afhopqrstu	1.96E-06
lmruvwx	7.64E-06	cfguvwxy	4.99E-06	buxy	2.91E-06	bopqrstu	1.95E-06
ikquvwxy	7.62E-06	iklnpuvwxy	4.99E-06	cfghopqrsxy	2.89E-06	ilmpuvwx	1.94E-06
deguvwx	7.62E-06	dfopqrstuv	4.98E-06	ghiopqrsty	2.88E-06	bcdefghuw	1.94E-06
ehiopqrst	7.54E-06	begx	4.92E-06	bdfhopqrs	2.85E-06	ilpuvwxy	1.90E-06
mnruvwx	7.47E-06	bdfopqrstu	4.89E-06	acdopqrstu	2.83E-06	mnopqrstuv	1.89E-06
morst	7.44E-06	hjlo	4.66E-06	degopqrstu	2.76E-06	efhopqrstu	1.86E-06
dfopqrstu	7.42E-06	bgiopqrt	4.64E-06	gnopqrstuv	2.76E-06	fnopqrstu	1.84E-06
gopqrx	7.38E-06	fgmnopqrstu	4.61E-06	beghiopqrs	2.74E-06	mopqrstuv	1.83E-06
fgquvwxy	7.28E-06	egry	4.59E-06	aghopqt	2.73E-06	efhuvwxy	1.77E-06
acdhuvwy	7.24E-06	adegopqrsty	4.47E-06	abcefghij	2.66E-06	gmopqrsu	1.75E-06
cefhopq	7.06E-06	befghux	4.46E-06	abfopqrsu	2.65E-06	fgmopqrstuv	1.74E-06
cioptuvwxy	7.05E-06	begxy	4.41E-06	ghmopqrstu	2.65E-06	adfghopqrst	1.71E-06
agopqrstu	6.88E-06	hjkoq	4.40E-06	jlr	2.64E-06	fhnopqrstuv	1.69E-06
beguvwx	6.83E-06	egpqrst	4.38E-06	arsxy	2.64E-06	fmopqrstu	1.69E-06
iklpuvwxy	6.78E-06	abegopqrt	4.30E-06	bgrx	2.58E-06	adgopqrstu	1.67E-06
agrsx	6.71E-06	acopqrsy	4.25E-06	opqst	2.52E-06	bdeqtu	1.64E-06
bcx	6.71E-06	bfopqrstuv	4.20E-06	buwxy	2.49E-06	er	1.64E-06
bcefmx	6.69E-06	eilxy	4.16E-06	bopqrstuv	2.48E-06	befmnx	1.62E-06
adegpqrs	6.65E-06	ru	4.15E-06	huvwx	2.42E-06	egx	1.60E-06
adegopqt	6.56E-06	imnopqrs	4.11E-06	tv	2.41E-06	afho	1.59E-06
dhuvwxy	6.49E-06	bgopqrstu	4.05E-06	giopqrt	2.40E-06	dopqrstu	1.59E-06
beuxy	6.49E-06	mr	4.02E-06	abdegopqrstu	2.40E-06	degopqrsty	1.55E-06
abcefghu	6.44E-06	oty	3.97E-06	fghoq	2.38E-06	dfghopqrst	1.54E-06
bopq	6.41E-06	gopqrsu	3.93E-06	gmnopqrstu	2.37E-06	bfmopqrstu	1.54E-06
ioptuvwxy	6.38E-06	acdu	3.93E-06	cfhopqrstu	2.36E-06	fk	1.49E-06
ilmnuvwxy	6.37E-06	bjnx	3.85E-06	fnopqrstuv	2.34E-06	cfuvwxy	1.45E-06
cioptuvwx	6.35E-06	arxy	3.83E-06	abcdefghjuw	2.33E-06	beopqrsu	1.41E-06
oprstu	6.34E-06	begopqrstuv	3.82E-06	mns	2.33E-06	abdefopqrstu	1.41E-06
fhq	6.34E-06	hop	3.80E-06	abcdeghj	2.33E-06	eopqrsx	1.40E-06
dfgopqrst	6.28E-06	abcdefghjuwx	3.79E-06	pqrstx	2.29E-06	gmopqrstuv	1.38E-06
eopqrstu	6.25E-06	hkq	3.78E-06	afh	2.29E-06	ahjklmns	1.35E-06
mrst	6.24E-06	ms	3.75E-06	abfhopqrstu	2.27E-06	fmnopqrstu	1.35E-06
jklu	6.18E-06	efgmopqrsu	3.64E-06	ejklmnr	2.27E-06	abduvwxy	1.30E-06
cdeghopq	6.17E-06	bny	3.64E-06	cop	2.27E-06	bdeopqrstu	1.30E-06
gopqrstuv	6.04E-06	beuwxy	3.60E-06	mnopqrst	2.25E-06	jklmw	1.23E-06
cdfhopqrstu	6.01E-06	adeguvwx	3.51E-06	ay	2.21E-06	adeuvwxy	1.23E-06
puv	5.61E-06	beopqrstuv	3.48E-06	bopqrstwy	2.17E-06	aopqrstu	1.23E-06
adx	5.59E-06	jpqrst	3.30E-06	bwxy	2.14E-06	bdeqtuv	1.23E-06
acfgh	5.52E-06	pt	3.26E-06	ehopqrstu	2.12E-06	ciopuvwxy	1.22E-06
pq	5.52E-06	iopqtuvwxy	3.25E-06	bdefqtuv	2.12E-06	ghopqrsty	1.21E-06
fgnopqrstuv	5.49E-06	nrsxy	3.21E-06	eghiopqrs	2.11E-06	abdeqtuv	1.21E-06
ghmo	5.48E-06	abdopqrstu	3.17E-06	abopqrsty	2.10E-06	fgmnopqrstuv	1.21E-06
pqr	5.48E-06	bdm	3.17E-06	abopqrstu	2.10E-06	aeqrtu	1.20E-06
ajsxy	5.37E-06	lqrs	3.16E-06	adix	2.06E-06	dehopqrstu	1.20E-06
hjklmns	5.34E-06	aeopqrst	3.13E-06	adopqrstuv	2.03E-06	fgquvwx	1.19E-06

Code	Frequency	Code	Frequency	Code	Frequency	Code	Frequency
abcefghux	1.16E-06	ciopqrst	6.62E-07	jr	4.04E-07	bdeoqtuv	2.46E-07
fmopqrst	1.16E-06	hmo	6.54E-07	buvwx	3.99E-07	lnrtx	2.45E-07
nrsx	1.16E-06	eklmnqwxy	6.52E-07	gmnopqrst	3.98E-07	adeqtuv	2.39E-07
bpqrs	1.14E-06	deqtu	6.38E-07	begux	3.86E-07	adgopqrstuv	2.37E-07
afgho	1.13E-06	abcdeqtuv	6.29E-07	cdeqtu	3.84E-07	lrstx	2.33E-07
nsxy	1.11E-06	rsy	6.20E-07	bcen	3.83E-07	asx	2.31E-07
rstx	1.08E-06	inouvwxy	6.18E-07	hlo	3.75E-07	fhjklmnos	2.27E-07
efgmnopqrstu	1.08E-06	defopqrstu	6.05E-07	begm	3.74E-07	bcdeqrtu	2.23E-07
bdefqtu	1.07E-06	cdeqtuv	6.02E-07	lnrx	3.70E-07	biopqrt	2.16E-07
hk	1.07E-06	fhopq	6.00E-07	bdeoqtu	3.57E-07	hmnopqrst	2.11E-07
klmnv	1.06E-06	cgopqrsu	5.93E-07	bdefopqrstuv	3.57E-07	dfhopqrsu	2.11E-07
eghiopqrsty	1.06E-06	defqtu	5.93E-07	fhmopqrst	3.51E-07	egtx	2.11E-07
dfghopqrs	1.03E-06	bcdeqtuv	5.90E-07	dhopqrstuv	3.39E-07	beix	2.10E-07
cfhuvwxy	1.03E-06	kmruvwx	5.80E-07	adefhopqrstu	3.35E-07	lrsx	2.07E-07
egopqrsu	1.03E-06	aqrtuv	5.80E-07	abcdetuv	3.31E-07	acdeqtu	2.06E-07
cfgopqrsy	1.02E-06	abdefqtuv	5.77E-07	abdopqrstuv	3.30E-07	befghuvwy	2.02E-07
ceghjopq	1.00E-06	eghiopqrsy	5.64E-07	fghuvwx	3.28E-07	lrtx	2.02E-07
bghiopqrst	1.00E-06	gmopqrst	5.57E-07	bdefqrtuv	3.27E-07	bdopqrstu	1.98E-07
dfhopqrstuv	1.00E-06	jpqrsxy	5.55E-07	adegopqrsu	3.25E-07	fgopqrst	1.97E-07
bopqrsw	9.82E-07	hjloq	5.53E-07	bux	3.24E-07	defoqtu	1.96E-07
abdefghj	9.34E-07	cdhopqrstu	5.50E-07	cfouvwxy	3.20E-07	klmnx	1.95E-07
aduvwxy	9.28E-07	hjkloq	5.43E-07	cdegopqrstu	3.10E-07	bcdeopqrstu	1.93E-07
cefghopqs	9.26E-07	abdefqtu	5.42E-07	nrstx	3.10E-07	cegopqrstx	1.91E-07
dfhopqrst	9.21E-07	cguvwxy	5.37E-07	abcdeopqrstu	3.06E-07	cfgopqrsxy	1.88E-07
efgmnopqrstuv	8.97E-07	couvwxy	5.36E-07	oqr	3.04E-07	opqrstuv	1.87E-07
bdefgopqrs	8.93E-07	copqrsx	5.23E-07	bgnopqrstu	3.04E-07	chopq	1.80E-07
hjoq	8.68E-07	dgopqrsty	5.23E-07	hkopq	3.04E-07	abgl	1.78E-07
acdopqrstuv	8.60E-07	bgmopqrstu	5.12E-07	defhopqrstuv	3.03E-07	abdefg	1.78E-07
hjklq	8.35E-07	fgopqrstx	5.06E-07	gpqrstx	3.02E-07	eoqrstuv	1.72E-07
fhjlo	8.34E-07	abdeqtu	4.95E-07	deopqrstu	2.99E-07	aefg	1.66E-07
bcop	8.33E-07	abegopqrstuv	4.90E-07	cfopqrsxy	2.97E-07	adefopqrstu	1.65E-07
fmopqrstuv	8.27E-07	defqtuv	4.86E-07	dfhmopqrstu	2.95E-07	hjkl	1.63E-07
bfnopqrstuv	8.26E-07	jmry	4.84E-07	kopqrsy	2.95E-07	bdetuv	1.63E-07
di	8.07E-07	ajrsxy	4.68E-07	ilmopquvwxy	2.87E-07	adegopqrstuv	1.62E-07
abefghj	8.06E-07	bcdeqtu	4.63E-07	bgl	2.87E-07	abdetuv	1.62E-07
nrx	7.93E-07	defqrtu	4.63E-07	acdefguvwx	2.82E-07	afopqrstuv	1.61E-07
lmv	7.82E-07	acdeguvwxy	4.63E-07	aeoqrstu	2.79E-07	ghopqrst	1.60E-07
bdefh	7.71E-07	lnrstx	4.59E-07	gnopqrsu	2.76E-07	abcdetu	1.59E-07
ahopqrstu	7.67E-07	bcdfgh	4.50E-07	bfgopqrstu	2.76E-07	afgh	1.57E-07
eqrstuv	7.63E-07	muvwx	4.47E-07	abfopqrstuv	2.76E-07	acdetuv	1.55E-07
ajklmn	7.60E-07	aeoqrtu	4.46E-07	fhjklq	2.69E-07	bgnopqrstuv	1.55E-07
hkloq	7.53E-07	acduwy	4.44E-07	gmopqrs	2.68E-07	nrtx	1.55E-07
defhopqrsu	7.22E-07	ioxy	4.43E-07	aopqrt	2.66E-07	btuvwxy	1.54E-07
fhjloq	7.19E-07	adeopqrst	4.36E-07	deqrtu	2.62E-07	adfgho	1.54E-07
bl	7.08E-07	adeopqrstu	4.36E-07	fgmopqrs	2.59E-07	ehiopqrsty	1.52E-07
aeopqrstu	7.08E-07	nopqrstuv	4.34E-07	bdeqrtu	2.58E-07	bopqrsx	1.52E-07
gi	6.92E-07	adfghopqrs	4.28E-07	abdeopqrstuv	2.54E-07	iklopuvwxy	1.49E-07
deqtuv	6.88E-07	fghopq	4.23E-07	knruvwx	2.49E-07	abdetu	1.48E-07
abcdeqtu	6.74E-07	aeqrtuv	4.22E-07	degopqrstuv	2.47E-07	aeoqrtuv	1.47E-07
aegopqrstu	6.66E-07	egrx	4.17E-07	bhiopqrst	2.46E-07	gos	1.43E-07

Code	Frequency	Code	Frequency	Code	Frequency	Code	Frequency
cfhouvwx	1.43E-07	abdeoqtu	6.15E-08	eopqrsu	2.04E-08	befuvwxy	6.98E-09
huw	1.42E-07	detu	6.13E-08	aegopqrt	2.03E-08	agh	6.92E-09
acfopqrstu	1.42E-07	egy	6.06E-08	iotxy	2.02E-08	knqrsxy	6.71E-09
deu	1.40E-07	abdgopqrsu	6.02E-08	bcdefg	2.01E-08	abcdeqrtu	6.56E-09
js	1.35E-07	acdeqtuv	6.00E-08	adefopqrstuv	1.85E-08	abcdefqtuv	6.48E-09
fu	1.34E-07	cdeopqrstu	5.95E-08	abcdx	1.84E-08	defopqrsu	6.34E-09
hr	1.31E-07	oq	5.73E-08	eijlx	1.83E-08	knqrsx	6.14E-09
bfgnopqrstu	1.29E-07	dghuvwxy	5.48E-08	chuvw	1.76E-08	aeqtu	6.05E-09
abdefopqrstuv	1.28E-07	ikopquvwxy	5.25E-08	bdertuv	1.73E-08	fhjklmno	6.02E-09
abeg	1.25E-07	cl	5.19E-08	cdfhopq	1.68E-08	juwxy	5.95E-09
dfhopqrs	1.24E-07	abdefqrtu	5.15E-08	begt	1.66E-08	abcdeoqtuv	5.90E-09
giuvwxy	1.17E-07	no	4.91E-08	abdeoqtuv	1.55E-08	fgh	5.64E-09
deqrtuv	1.17E-07	acdhopqrstu	4.86E-08	bdh	1.50E-08	hjopqs	5.40E-09
bsx	1.14E-07	auvwx	4.75E-08	acd	1.31E-08	bmopqrstu	5.38E-09
adeqtu	1.09E-07	abdefoqtu	4.72E-08	jkuwxy	1.30E-08	afgnopqrstuv	5.09E-09
gopqrsty	1.07E-07	aeopqrstx	4.65E-08	eps	1.29E-08	iklopquvwxy	5.01E-09
bdefoqtu	1.06E-07	qx	4.64E-08	bcdefopqrstu	1.26E-08	uwx	4.97E-09
aegopqrstuv	1.06E-07	dgopqrstu	4.55E-08	cdeqrtu	1.19E-08	abdeopqrsu	4.87E-09
abg	1.05E-07	fopqrst	4.36E-08	agopqrstuv	1.19E-08	bgr	4.84E-09
beiopqrs	1.04E-07	ei	4.18E-08	bcefg	1.19E-08	begopqrstxy	4.62E-09
adgopqrt	1.04E-07	bcdeoqtuv	4.14E-08	cdfhopqrstuv	1.18E-08	qru	4.26E-09
bcdh	1.03E-07	klruvwx	4.14E-08	abdefopqrsu	1.18E-08	bcdetuv	4.18E-09
dg	1.02E-07	ijotxy	3.88E-08	cemn	1.18E-08	knqruvwx	3.85E-09
befguvwy	1.02E-07	befopqrstu	3.87E-08	abefghw	1.17E-08	abeghuw	3.49E-09
adefqtu	1.01E-07	mnrtx	3.76E-08	eopqrstuv	1.15E-08	knrsxy	3.37E-09
detuv	1.00E-07	adoqrtu	3.62E-08	deftu	1.14E-08	hnopqrstxy	3.35E-09
lmrtx	9.88E-08	fhjl	3.44E-08	hjklo	1.14E-08	ehuvwxy	3.28E-09
bdefoqtuv	9.63E-08	bcdeqrtuv	3.41E-08	adoqrstuv	1.13E-08	adeoqrtuv	3.26E-09
fghopqrsxy	9.57E-08	bdefotuv	3.31E-08	acdegopqrstu	1.12E-08	ilmopqrst	3.18E-09
fhjo	9.49E-08	gjk	3.26E-08	nr	1.11E-08	abceghij	3.06E-09
bdeftu	9.07E-08	bdeopqrstuv	3.19E-08	beqtu	1.07E-08	cdfx	2.82E-09
abgopqrstuv	8.93E-08	amy	2.97E-08	dfgopqrstu	1.05E-08	abeoqrtu	2.79E-09
cegopqrstu	8.75E-08	adefqtuv	2.93E-08	aqrtu	1.03E-08	abdertuv	2.76E-09
bdefopqrsu	8.55E-08	kl	2.86E-08	ceh	1.02E-08	eoqtuv	2.70E-09
kqrsxy	8.39E-08	defopqrstuv	2.73E-08	egmnopqrstuv	1.01E-08	fhopqrs	2.62E-09
hkoq	8.10E-08	mqy	2.63E-08	ijkotxy	1.01E-08	deghopqs	2.60E-09
bfgnopqrsu	8.04E-08	dfgopqrs	2.62E-08	impuvwx	9.84E-09	ewx	2.47E-09
bdfopqrstuv	8.00E-08	impuvwxy	2.53E-08	cdeo	9.82E-09	abcfghij	2.46E-09
abcdertu	7.92E-08	lm	2.53E-08	deqt	9.75E-09	abdgopqrstuv	2.39E-09
fghopqs	7.76E-08	fmopqrs	2.32E-08	bdefqrtu	9.72E-09	cegopqrstuv	2.26E-09
ijoxy	7.67E-08	beghopqrst	2.30E-08	am	9.59E-09	fghmopqst	2.25E-09
bdeftuv	7.65E-08	acdfhopqrstu	2.29E-08	dehopqrstuv	9.16E-09	deoqtuv	2.14E-09
adgopqrsu	7.57E-08	cfhopq	2.29E-08	hjl	9.10E-09	lrx	2.12E-09
bdetu	7.49E-08	defoqtuv	2.27E-08	bdx	7.96E-09	jopqrsx	2.10E-09
abxy	7.28E-08	defhmopqrstu	2.20E-08	bchi	7.61E-09	cimopqrst	1.89E-09
cfg	6.91E-08	hjkq	2.17E-08	gmopqrstx	7.53E-09	bfg	1.85E-09
dfghopqrstu	6.47E-08	ko	2.17E-08	acdeopqrstu	7.23E-09	bcefghuwx	1.65E-09
hlopqrstu	6.44E-08	bdeopqrsu	2.16E-08	qrsxy	7.18E-09	chmuvw	1.53E-09
mnqruvwx	6.43E-08	abdfopqrstuv	2.11E-08	vwy	7.03E-09	ijtxy	1.47E-09
abdefotuv	6.35E-08	abdopqrst	2.05E-08	adfhopqrstu	7.01E-09	acdefopqrstu	1.45E-09

Code	Frequency	Code	Frequency	Code	Frequency	Code	Frequency
hopqrstuv	1.45E-09	jklmno	3.85E-10	befgopqrstu	5.94E-11	cgmopqrstu	5.89E-12
aeguvwxy	1.33E-09	adeopqrstuv	3.73E-10	bdefotu	5.65E-11	acixy	5.69E-12
achjuvwy	1.32E-09	jkuwx	3.69E-10	cdetuv	5.45E-11	egxy	5.69E-12
hlmopqrst	1.26E-09	dehuvwxy	3.57E-10	abefopqrstuv	5.29E-11	defgh	5.26E-12
ikopuvwxy	1.22E-09	aoqrstuv	3.49E-10	acdopqrsy	5.25E-11	bdefoqstu	4.73E-12
defgopqrstu	1.14E-09	elxy	3.49E-10	nopqrsxy	4.92E-11	bdfgh	4.70E-12
begu	1.13E-09	ghiopqrs	3.45E-10	aeqrstu	4.75E-11	egpqrsx	4.52E-12
abfgnopqrstu	1.12E-09	abefghu	3.36E-10	cdefopqrstu	4.75E-11	bdegopqrstu	4.51E-12
jkuxy	1.11E-09	cjk	3.10E-10	qrsx	4.66E-11	ciopuvwx	4.26E-12
bcdeghj	1.08E-09	bfmopqrstuv	3.06E-10	fhquvwxy	4.57E-11	lpqrs	4.01E-12
abcdefopqrstu	1.05E-09	acdfg	3.04E-10	adfhopqrst	4.40E-11	efgnopqrstuv	3.37E-12
abdegopqrsu	9.98E-10	abcdeoqtu	3.04E-10	defqrtuv	4.36E-11	abfgnopqrstuv	3.14E-12
iklquvwxy	9.83E-10	abcdefghx	3.03E-10	afguvwx	4.31E-11	sy	3.09E-12
bfhopqrstuv	9.73E-10	fghquvwxy	3.01E-10	begh	3.86E-11	ghno	2.83E-12
bcdefghjuwx	9.66E-10	fhnopqrstu	2.92E-10	lmquvwx	3.43E-11	qrtu	2.50E-12
ahuvwy	9.66E-10	bmopqrstuv	2.90E-10	abfgopqrstuv	3.16E-11	bdefhuvwxy	2.49E-12
cfmopqrstu	9.60E-10	aqrstu	2.87E-10	abcdeg	2.91E-11	aeortuv	2.49E-12
acduy	9.60E-10	abcdeopqrstuv	2.66E-10	abcfh	2.57E-11	fgmnopqrst	2.46E-12
ikuvwx	9.37E-10	abcdeotu	2.54E-10	deoqtu	2.42E-11	fgopqrsu	2.39E-12
defu	9.14E-10	fjkln	2.30E-10	opt	2.16E-11	fgmopqrst	2.28E-12
clopqrsy	8.92E-10	ioqrst	2.28E-10	fgrsx	2.06E-11	bfgopqrstuv	2.18E-12
bfgmopqrstuv	8.79E-10	bdeqrtuv	2.16E-10	afgopqrstuv	1.85E-11	abfgopqrstu	2.15E-12
ijxy	8.73E-10	bdeqstu	2.04E-10	afgopqrstu	1.84E-11	fgmopqst	2.14E-12
abcdefghwx	8.00E-10	fuvwx	1.99E-10	acdwy	1.80E-11	dgopqrs	2.08E-12
abgrsx	7.88E-10	fghq	1.91E-10	gijklmny	1.72E-11	begopqrsty	2.04E-12
adguvwxy	7.32E-10	jkn	1.76E-10	abdegopqrstuv	1.72E-11	bhopqrstxy	2.00E-12
adegopqrstx	6.44E-10	defopqrst	1.74E-10	hopqs	1.69E-11	acefguvwx	1.96E-12
ikpquvwxy	6.26E-10	ghuvwxy	1.65E-10	adefoqtuv	1.61E-11	cdopqrstu	1.60E-12
cx	6.00E-10	bnopqrstu	1.60E-10	hjk	1.61E-11	abdeqrtuv	1.55E-12
acfhopqrstu	5.82E-10	aeopqrstuv	1.59E-10	gjkl	1.41E-11	bfghmopqrstuv	1.52E-12
adeoqrtu	5.78E-10	ghopqst	1.46E-10	bego	1.40E-11	agopqrty	1.07E-12
hjq	5.78E-10	jkux	1.41E-10	fguvwx	1.36E-11	ilmopqrs	1.06E-12
adfgopqrstuv	5.67E-10	cdegopqrstuv	1.37E-10	abdefguvwxy	1.31E-11	bfhmopqrstuv	1.03E-12
binx	5.44E-10	ijklmny	1.33E-10	kux	1.30E-11	deo	9.67E-13
aegopqrs	5.39E-10	klmv	1.23E-10	efhopqrsu	1.29E-11	bopqrswy	9.49E-13
efgqu	5.35E-10	kuxy	1.22E-10	hjklmnos	1.27E-11	ns	9.11E-13
bcefghjuw	5.26E-10	kuwxy	1.20E-10	abcdefopqrstuv	1.25E-11	abeopqrsx	6.12E-13
deopqrstuv	5.18E-10	dfgopqrstuv	1.14E-10	abdefghjuwx	1.24E-11	aeopqrt	5.98E-13
adfgopqrstu	5.09E-10	ceghopqs	1.01E-10	aopqrstxy	1.11E-11	jm	5.40E-13
gmnopqrstuv	5.02E-10	abdeftuv	9.75E-11	copqrstuv	1.09E-11	abfuvwx	4.95E-13
bcegy	4.81E-10	bilxy	9.54E-11	adfuvwxy	9.18E-12	beux	4.66E-13
quv	4.78E-10	egiopqrs	8.84E-11	adgpqrs	9.10E-12	lmtx	4.27E-13
befmx	4.45E-10	beory	8.46E-11	bcemn	8.22E-12	abfhopqrstuv	4.11E-13
cdegopq	4.44E-10	opqrstw	8.18E-11	deh	7.44E-12	deftuv	3.60E-13
bfhmopqrstu	4.35E-10	mrx	7.79E-11	abefopqrstu	7.22E-12	bdefoqrtuv	3.45E-13
adeg	4.30E-10	luvwx	7.50E-11	euwxy	7.18E-12	fhmopqrstuv	3.40E-13
acdegopqrstuv	4.15E-10	ijnouvwxy	7.49E-11	klmuvwx	6.87E-12	defoqt	3.21E-13
eghopqrst	4.14E-10	hox	7.41E-11	abcefghuw	6.85E-12	abfgnopqrst	2.92E-13
fhjklmn	3.95E-10	abdertu	6.46E-11	bewxy	6.77E-12	bcdeopqrsu	2.74E-13
bdefopqrst	3.92E-10	bd	6.19E-11	bdefhopqrstu	6.55E-12	lntx	2.55E-13

Code	Frequency	Code	Frequency	Code	Frequency	Code	Frequency
efhuvwy	2.39E-13	aertu	9.69E-16	abcopqrsu	4.97E-18	abcdeghuwx	1.21E-20
bcdfx	2.39E-13	fghjo	6.95E-16	pwy	3.76E-18	hmopqrstuv	1.16E-20
abdefhopqrstu	2.32E-13	abcdeghij	6.90E-16	nopqrstwy	2.78E-18	ajxy	1.16E-20
efgopqs	2.14E-13	adeorstu	6.66E-16	klqruvwx	2.63E-18	abgopqrt	1.09E-20
bu	2.07E-13	cdeoqtuv	6.47E-16	agopqrstx	1.86E-18	acdopqrst	1.04E-20
mrtx	2.06E-13	eghopqs	5.86E-16	aeoqtu	1.75E-18	cfhuvwx	6.30E-21
deotuv	1.94E-13	cfhmopqrstu	5.49E-16	egmopqrt	1.65E-18	bmy	6.04E-21
ilmnpuvwxy	1.85E-13	agopqrt	5.39E-16	abceh	1.19E-18	defoqrtuv	3.84E-21
fghlopqrs	1.83E-13	abcdgh	5.33E-16	abcdefopqrsu	1.14E-18	ewxy	3.49E-21
bijnx	1.70E-13	bghopqrst	4.01E-16	iklpquvwxy	1.09E-18	bcmny	3.09E-21
cdiu	1.42E-13	abdefoqrstu	3.77E-16	cdefghopqs	1.04E-18	jopqrsy	2.26E-21
adefqrtu	1.41E-13	ejkuwxy	3.61E-16	copq	9.36E-19	bcdefopqrstuv	2.01E-21
bfghmopqrstu	1.35E-13	abcdopqrstu	3.50E-16	abcefgj	8.18E-19	abcdeqrtuv	1.44E-21
iklmnquvwxy	9.88E-14	adefuvwxy	2.95E-16	abdeotuv	8.10E-19	bgopqrsx	8.50E-22
bcopqrstuv	6.95E-14	msxy	2.26E-16	fghjoq	6.87E-19	ijnopuvwxy	7.79E-22
defghjopq	6.46E-14	efgh	2.21E-16	fhuvwy	6.39E-19	juxy	7.19E-22
abdefopqrst	6.31E-14	mno	1.82E-16	abefg	6.24E-19	do	6.02E-22
dfhuvwx	4.99E-14	fgopqrstxy	1.81E-16	mot	5.76E-19	chuvwx	4.37E-22
abceg	4.55E-14	adijkln	1.77E-16	loqrs	4.12E-19	bdeopqrst	2.66E-22
bfgnopqrstuv	4.38E-14	cegjopq	1.65E-16	fhmn	3.86E-19	efgopqrstu	2.65E-22
cfghouvwxy	3.68E-14	ijkoxy	1.55E-16	egpqrs	3.78E-19	deopqrsuv	2.48E-22
degopqt	3.01E-14	mnx	1.29E-16	tx	3.10E-19	aegopqrstx	2.17E-22
abeqtuv	2.75E-14	abdeqrtu	1.14E-16	beguxy	2.86E-19	ce	1.31E-22
eqrtuv	2.30E-14	egi	1.01E-16	os	2.39E-19	oqrstu	1.26E-22
fghmopqrstu	2.21E-14	adefopqrsu	9.97E-17	kqrsx	2.21E-19	cdefghjopqs	1.14E-22
nrst	2.15E-14	begopqrstx	9.53E-17	ade	1.79E-19	kmnv	1.07E-22
ikmpuvwxy	2.01E-14	aouvwx	8.57E-17	fmnopqrstuv	1.38E-19	bcdeopqrstuv	9.87E-23
fhjkl	1.87E-14	biy	7.21E-17	fquvwxy	1.33E-19	hmopqrstu	6.07E-23
qw	1.69E-14	ehopqrstuv	7.16E-17	ejklm	1.32E-19	abfiuvwx	4.50E-23
dfghopqrsu	1.38E-14	gjopqrsxy	5.89E-17	eopq	1.21E-19	iouvwx	3.34E-23
abeoqrstu	1.17E-14	eklmnqwx	5.55E-17	beuvwy	1.15E-19	cdgh	3.18E-23
adfgopqrsu	9.36E-15	befghuvwxy	5.51E-17	dfhmopqrstuv	1.12E-19	cdf	3.18E-23
cfgnopqrstx	8.47E-15	fhmnopqrstu	5.47E-17	abegiopqrstw	1.02E-19	bilx	3.00E-23
fgmopqrsu	7.70E-15	bcdefghjuw	4.22E-17	abdgopqrsuv	9.30E-20	fopqrsu	2.87E-23
aeqtuv	7.29E-15	cf	3.81E-17	acefgh	7.70E-20	begrsx	2.67E-23
adqrstu	7.10E-15	abefguvwxy	3.76E-17	bgry	7.44E-20	bhopqrst	2.55E-23
cefg	5.49E-15	knqrvwx	3.67E-17	aoqrstu	7.32E-20	aeu	2.53E-23
gmno	5.07E-15	abdefqrtuv	2.77E-17	cgiopqrst	7.30E-20	cr	2.45E-23
agrx	4.98E-15	efgmopqrstu	2.71E-17	befhuvwy	7.15E-20	ajrxy	2.42E-23
efgmopqrstuv	4.30E-15	dhuvwy	2.23E-17	qsxy	5.67E-20	osuvwx	2.35E-23
iopqrsy	3.99E-15	eklqwxy	1.73E-17	optuvwx	5.14E-20	aeoqrstuv	1.82E-23
efghjopq	3.79E-15	fhjkoq	1.69E-17	cmopqrstu	4.50E-20	egjklmn	1.54E-23
cfghopqs	3.35E-15	cefghopq	1.64E-17	bdefmx	4.48E-20	qrvwx	1.32E-23
beilx	2.91E-15	behuvwy	1.53E-17	aortuv	3.62E-20	fgmnopqrs	1.32E-23
defgopqrst	2.56E-15	degopqrs	1.40E-17	adfopqrstuv	3.01E-20	giopqrsty	1.12E-23
mopqrsxy	2.46E-15	befguvwxy	1.03E-17	bjkl	2.43E-20	vy	6.10E-24
ciuvwxy	2.02E-15	ijkxy	1.01E-17	inopuvwxy	1.93E-20	biuvwxy	5.12E-24
bcdeotu	1.85E-15	bopqrtx	9.58E-18	cefghjopqs	1.76E-20	adfgopqrs	3.33E-24
eklnqwxy	1.44E-15	degopqrsu	9.11E-18	bcdetu	1.72E-20	exy	2.98E-24
adeqrtuv	9.81E-16	aorstu	8.51E-18	begopqrsx	1.33E-20	rtx	2.85E-24

Code	Frequency	Code	Frequency	Code	Frequency	Code	Frequency
acix	2.29E-24	cde	7.49E-30	abefgu	2.15E-40	dfhu	1.39E-56
cjklmnx	2.06E-24	mtx	2.05E-30	ghrx	1.17E-40	bgiopqrs	9.41E-58
jxy	1.71E-24	bdeoqrtu	1.83E-30	hpq	9.15E-41	vwxy	6.21E-58
abdeuvwxy	1.39E-24	adgopqr	1.29E-30	cehjopqs	4.19E-41	bdegopqrsx	9.14E-59
ltx	1.38E-24	egors	1.13E-30	ajklm	3.32E-41	eqtuv	6.33E-59
fghopqt	1.02E-24	agopqt	1.10E-30	ghopqrsx	3.08E-41	cdjuvwy	2.88E-59
cdopqrstuv	8.31E-25	kuwx	1.04E-30	befuvwy	2.32E-41	begwxy	2.69E-59
cegjopqs	8.26E-25	bcefghi	9.49E-31	bfhuvwy	8.70E-42	beqtuv	2.63E-59
behiopqrst	8.23E-25	cefguvwxy	2.94E-31	egt	3.51E-42	fnopqrsu	2.42E-59
abdfu	4.65E-25	mnv	2.68E-31	efghopqs	2.57E-42	adm	1.01E-59
bdefhopqrstuv	4.41E-25	lmnquvwx	2.63E-31	befhuvwxy	2.11E-42	aby	1.10E-60
adgpqrst	2.39E-25	biopqr	8.78E-32	hjklm	1.20E-42	uwy	5.08E-61
cdehopq	1.67E-25	aboqrtu	6.66E-32	abcdefghij	8.35E-43	bdeoqt	1.24E-62
jklmns	1.43E-25	aeorstu	6.22E-32	gpqrs	3.98E-43	gquvwxy	1.21E-62
acdopqrsu	1.36E-25	gpqrst	4.65E-32	gjopqrsy	1.98E-43	adefqrtuv	2.97E-64
aixy	1.19E-25	dps	4.05E-32	adefopqrsuv	1.54E-43	qsy	2.78E-64
jopqrst	9.19E-26	abeopqrt	3.95E-32	abeoqrtuv	1.35E-43	jkx	9.12E-67
adegopqrsuv	6.97E-26	grs	3.54E-32	hiquvwxy	6.02E-44	hkl	1.79E-67
buwx	6.83E-26	mqrst	3.28E-32	acfh	3.22E-44	bgnopqrst	1.01E-68
bcm	3.66E-26	bmny	2.74E-32	fhu	1.82E-44	anrsx	6.97E-69
ghopqrstu	3.23E-26	bdefhopqrs	1.88E-32	mnopqrsu	1.29E-44	eopqrsy	1.47E-71
jklmnuw	1.48E-26	befghuvw	7.72E-33	acjuvwy	1.01E-44	bjn	5.18E-73
gnopqrst	9.32E-27	abcdeu	5.70E-33	kxy	9.89E-45	cpu	5.33E-75
fhlopqrsu	8.17E-27	beghuvwxy	1.81E-33	ef	8.65E-45	befghi	8.45E-79
jlxy	6.10E-27	cdfh	1.29E-33	abgopqrsu	6.82E-45	abgrx	2.18E-79
abdegopqrsuv	3.65E-27	bfhopqrstu	1.18E-33	hjklmnt	3.69E-45	fhmnopqrst	2.92E-80
aortu	1.85E-27	bceghij	1.07E-33	dex	3.23E-45	prsv	1.18E-80
bgiopqrsy	1.81E-27	krsxy	6.84E-34	egmopqrstu	8.63E-46	afgmopqt	1.11E-81
fghouvwxy	1.60E-27	befqtuv	5.86E-34	klv	2.03E-47	bdegopqrstuv	5.18E-82
fhuvwx	1.23E-27	hjklmnr	2.95E-34	ikmnquvwxy	1.48E-47	uy	2.68E-84
flopqrs	1.10E-27	ituvwxy	2.28E-34	hiopqrs	7.43E-48	deghouvwxy	4.86E-85
mpqrst	1.04E-27	bcmn	2.20E-34	cfgopqrstx	4.33E-48	eiuvwxy	8.37E-87
acdefghw	8.88E-28	cdefhopq	3.54E-35	vwx	2.56E-48	bemny	4.41E-88
cdefg	6.76E-28	mntx	3.13E-35	clopqrs	1.62E-48	fghmopqrs	1.74E-89
egopqrt	4.43E-28	fghopqrsy	1.21E-35	bmr	7.81E-49	bfgnopqrst	2.10E-90
abgopqr	3.40E-28	bfqu	2.83E-36	deghopq	2.79E-50	abeqrtuv	3.54E-92
ghiquvwxy	2.92E-28	jkwxy	2.67E-36	opq	2.44E-50	fs	6.51E-94
lmntx	2.45E-28	ciptuvwxy	2.44E-36	efgnopqrstu	6.86E-51	cdhopqrstuv	7.79E-95
abcdefoqtu	2.04E-28	opqs	1.84E-36	acfghopqrstuv	2.95E-51		
chjopqs	1.59E-28	fghj	1.34E-36	euvwx	2.53E-51		
cgopq	1.08E-28	aboqrtuv	4.97E-37	uw	1.37E-51		
bfh	3.66E-29	adfguvwx	3.95E-37	psv	6.97E-52		
nstx	3.36E-29	aeuvwxy	5.16E-38	abcefghjuwx	2.82E-52		
abdfhopqrstu	1.71E-29	iknuvwxy	3.11E-38	hjklmno	1.81E-52		
abcdeopqrsu	1.49E-29	esx	1.31E-38	lmnqruvwx	2.13E-53		
ghr	1.27E-29	afghopqrs	1.21E-38	bcdeghi	5.91E-55		
bhuvwy	9.55E-30	beij	4.56E-39	opqrswxy	4.93E-55		
beiq	8.32E-30	egmx	3.05E-39	opqrstwxy	3.48E-56		
abjxy	8.29E-30	afghmopqt	8.41E-40	abdx	2.14E-56		
abfqu	7.54E-30	cdex	4.89E-40	mxy	1.74E-56		
